# Supplementary material for: Threat management priorities for conserving Antarctic biodiversity
Source: PLoS Biol. 2022 Dec 22;20(12):e3001921. doi: 10.1371/journal.pbio.3001921 (PMC9778584; doi:10.1371/journal.pbio.3001921)
Supplement: S1 Text — File contains Figs A–F and Tables A–N. (PDF) [file pbio.3001921.s003.pdf]

S1 Text:

Supporting information for *'Threat management priorities for conserving Antarctic biodiversity'*

Jasmine R. Lee*, Aleks Terauds, Josie Carwardine, Justine D. Shaw, Richard A. Fuller, Hugh P. Possingham, Steven L. Chown, Peter Convey, Neil Gilbert, Kevin A. Hughes, Ewan McIvor, Sharon A. Robinson, Yan Ropert-Coudert, Dana M. Bergstrom, Elisabeth M. Biersma, Claire Christian, Don A. Cowan, Yves Frenot, Stéphanie Jenouvrier, Lisa Kelley, Michael J. Lee, Heather J. Lynch, Birgit Njåstad, Antonio Quesada, Ricardo M. Roura, E. Ashley Shaw, Damon Stanwell-Smith, Megumu Tsujimoto, Diana H. Wall, Annick Wilmotte, Iadine Chadès

*** Jasmine R Lee**

Email: jasmine.lee1@uqconnect.edu.au

This PDF file includes:

Figures A - F

Tables A - N

Other supporting information for this manuscript include the following:

S1 and S2 Data

Supporting Figures

a

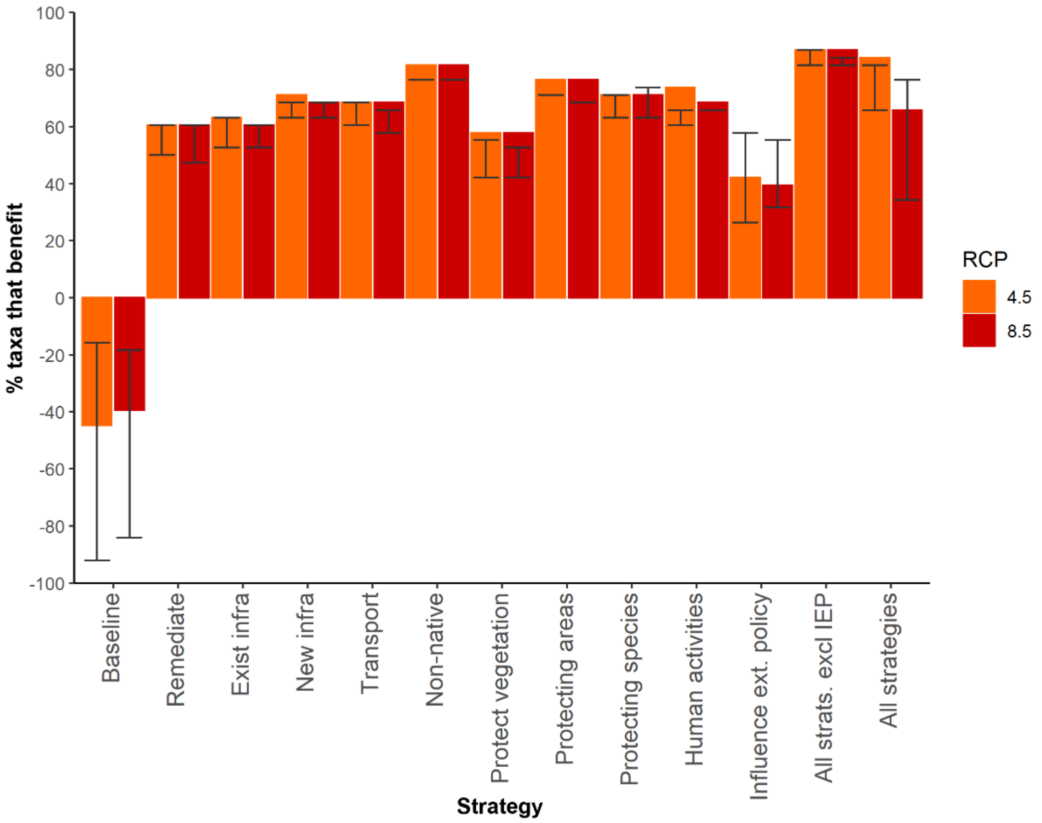

b

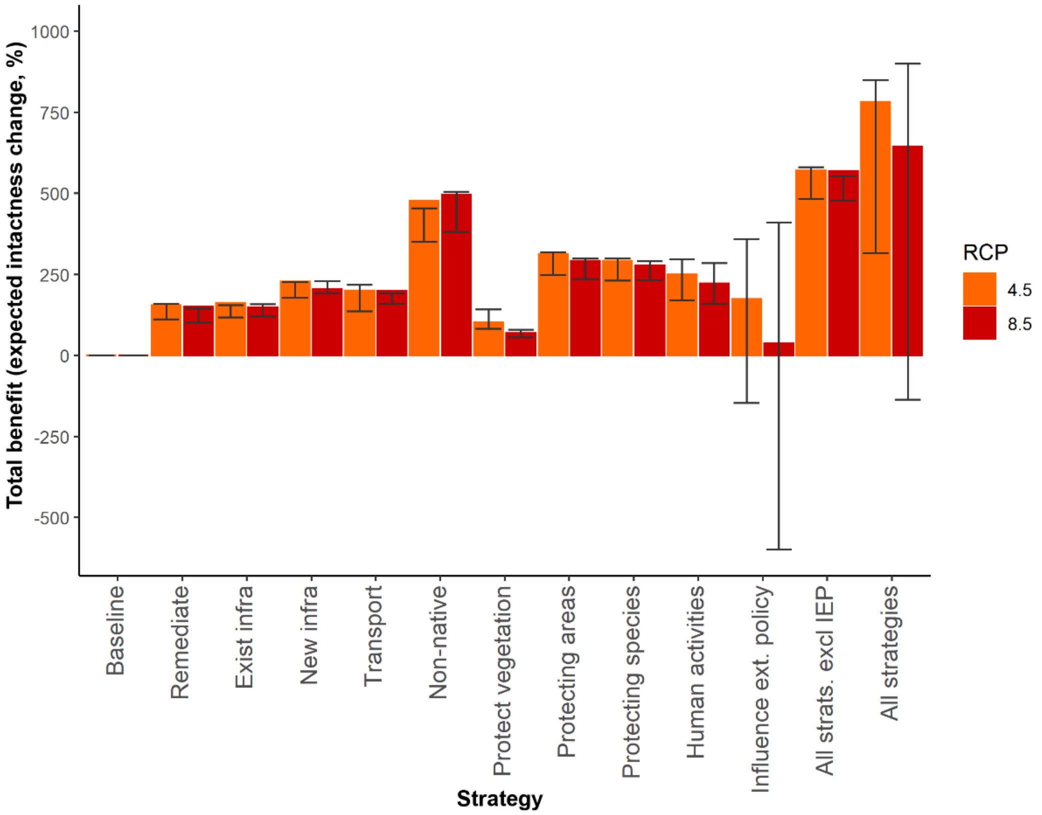

Figure A. Response of Antarctic terrestrial biodiversity to various conservation management strategies by the end of 2100 under two climate forcing scenarios (RCP4.5, RCP8.5). **a,** Percentage of taxa likely to benefit. **b,** Expected total benefit of strategies summed for all taxa and both regions combined. Bars represent the experts' best estimates when assessing benefit, whilst error bars represent upper (best case scenario) and lower (worst case scenario) bounds. An outcome of the *'Influence external policy* (IEP) and *'All strategies combined'* strategies is that carbon emissions are reduced globally (in line with the milder RCP2.6); however, benefits are still calculated relative to the baselines of RCP4.5 and RCP8.5. Values used to calculate benefit include benefits to taxa expected to expand beyond current (100%) intactness (An2). The data underlying this Figure can be found in S2 Data.

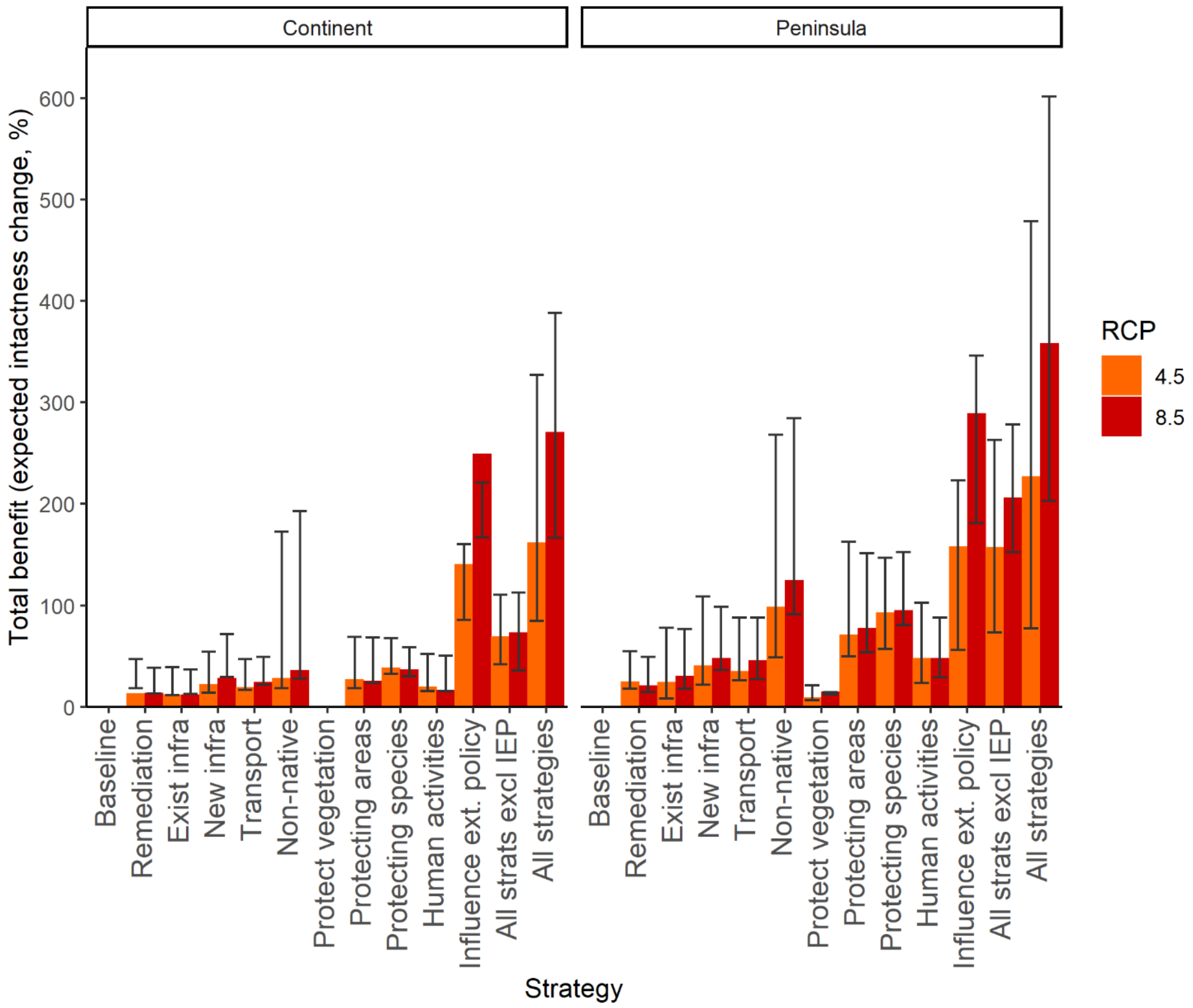

Figure B. Expected total benefit by region (continent and peninsula) of various conservation management strategies summed for all Antarctic taxa combined, at the end of 2100 under two climate forcing scenarios (RCP4.5, RCP8.5). Bars represent the experts' best estimates when assessing benefit, whilst error bars represent upper (best case scenario) and lower (worst case scenario) bounds. An outcome of the *'Influence external policy'* (IEP) and *'All strategies combined'* strategies is that carbon emissions are reduced globally (in line with the milder RCP2.6); however, benefits are still calculated relative to the baselines of RCP4.5 and RCP8.5. Values used to calculate benefit were capped at current (100%) intactness (An1). The data underlying this Figure can be found in S2 Data.

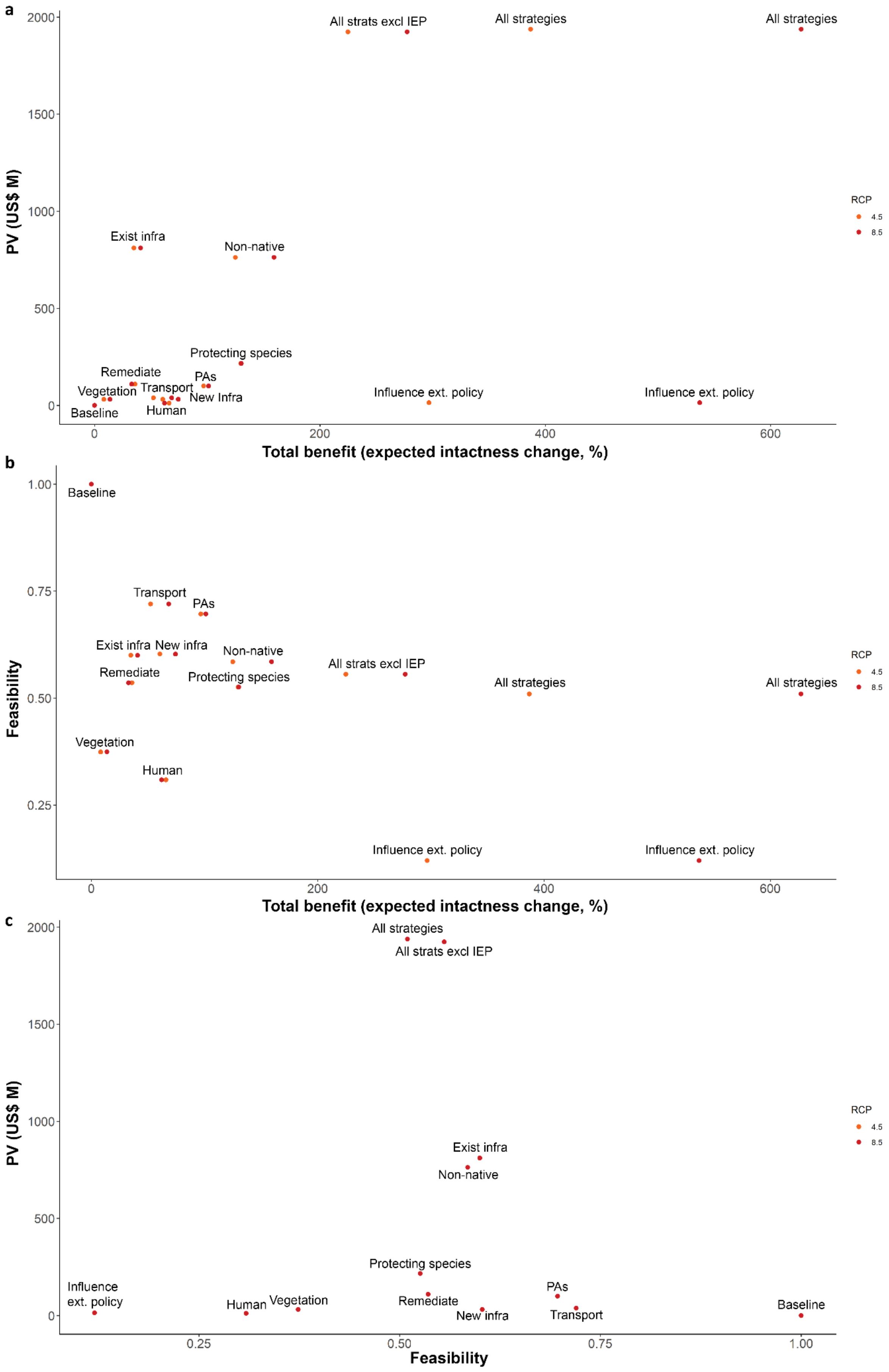

a
PV (US$ M)
2000
1500
1000
500
0
All strats excl IEP
All strategies
All strategies
Exist infra
Non-native
Protecting species
Remediate
PAs
Vegetation
Transport
New Infra
Influence ext. policy
Influence ext. policy
Baseline
Human
0
200
400
600
Total benefit (expected intactness change, %)
RCP
4.5
8.5
b
Feasibility
1.00
0.75
0.50
0.25
Baseline
Transport
PAs
Exist infra
New infra
Non-native
All strats excl IEP
Remediate
Protecting species
All strategies
All strategies
Vegetation
Human
Influence ext. policy
Influence ext. policy
0
200
400
600
Total benefit (expected intactness change, %)
RCP
4.5
8.5
c
PV (US$ M)
2000
1500
1000
500
0
All strategies
All strats excl IEP
Exist infra
Non-native
Protecting species
Influence
ext. policy
PAs
Human
Vegetation
Remediate
Baseline
New infra
Transport
0.25
0.50
0.75
1.00
Feasibility
RCP
4.5
8.5

Figure C. Expected benefit, estimated cost and feasibility of various conservation management strategies for Antarctic biodiversity by the end of 2100 under two climate forcing scenarios (RCP4.5, RCP8.5). **a,** Expected total benefit and estimated present value (PV), **b,** Expected total benefit and estimated feasibility, **c,** Estimated feasibility and estimated present value. Expected total benefit is summed for all taxa and both regions combined and is calculated using biodiversity experts' best estimates when assessing benefit. An outcome of the *'Influence external policy'* (IEP) and *'All strategies combined'* strategies is that carbon emissions are reduced globally (in line with the milder RCP2.6); however, benefits are still calculated relative to the baselines of RCP4.5 and RCP8.5. Values used to calculate benefit were capped at current (100%) intactness (An1). The data underlying this Figure can be found in S2 Data.

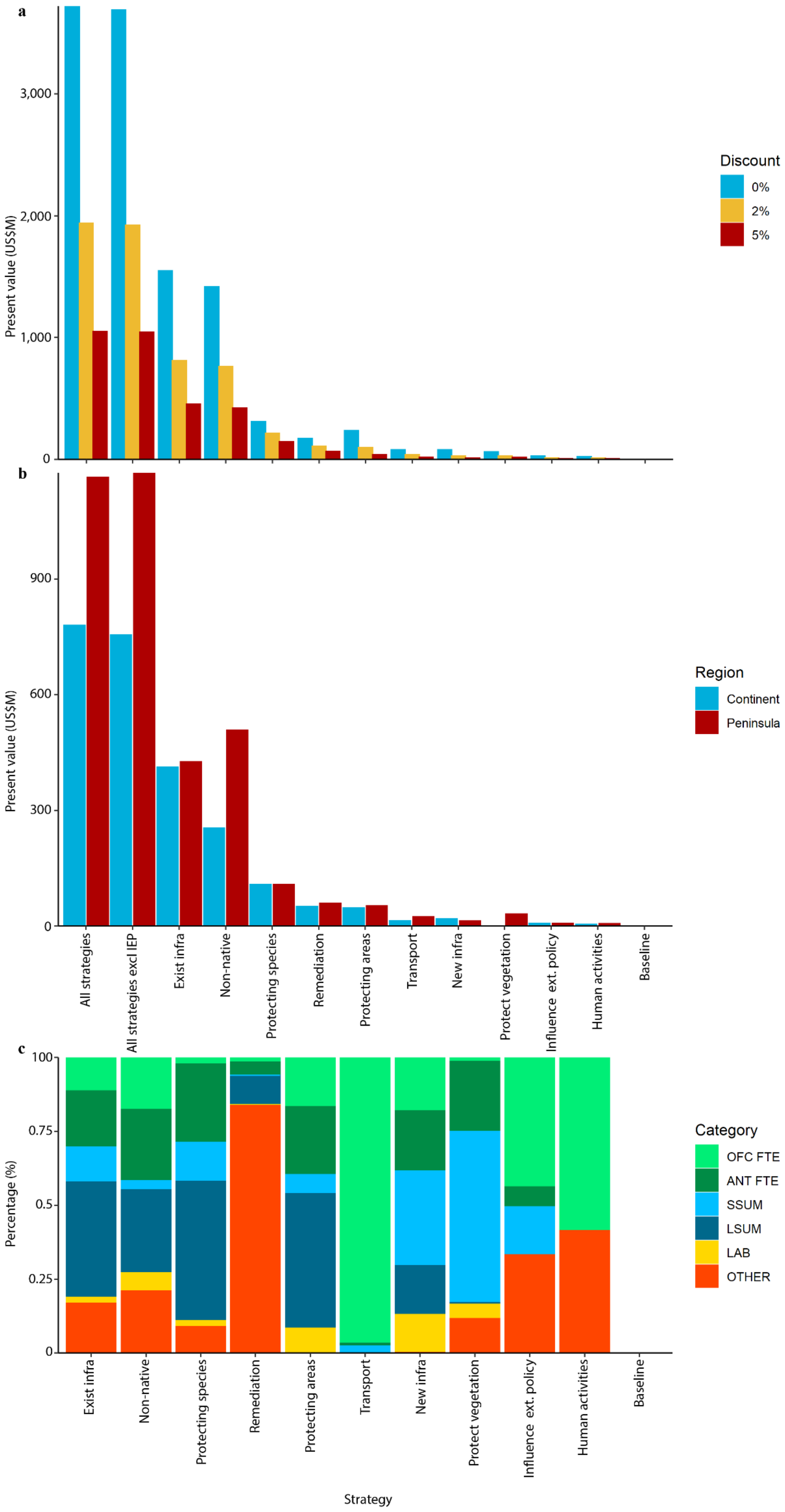

a
Present value (US$M)
3,000
2,000
1,000
0
Discount
0%
2%
5%
b
Present value (US$M)
900
600
300
0
Region
Continent
Peninsula
All strategies
All strategies excl IEP
Exist infra
Non-native
Protecting species
Remediation
Protecting areas
Transport
New infra
Protect vegetation
Influence ext. policy
Human activities
Baseline
c
Percentage (%)
100
0.75
0.5
0.25
0
Category
OFC FTE
ANT FTE
SSUM
LSUM
LAB
OTHER
Exist infra
Non-native
Protecting species
Remediation
Protecting areas
Transport
New infra
Protect vegetation
Influence ext. policy
Human activities
Baseline
Strategy

Figure D. Cost comparison and contribution to costs of various conservation management strategies for Antarctic biodiversity. **a,** Estimated present value using three different social discount rates. Ordered from most to least expensive using a 2% discount rate. **b,** Estimated present value across two different regions. **c,** Percentage breakdown of primary cost items for various conservation management strategies for Antarctic biodiversity. OFC FTE – Office full-time equivalent employees, ANT FTE – Antarctic based full-time equivalent employees, SSUM – short summer Antarctic berths (6 weeks), LSUM Antarctic berths (3 months), LAB – lab analysis costs post field sample collection per event, OTHER – other costs, such as captive breeding programs, remediation, flights, combined. The data underlying this Figure can be found in S2 Data.

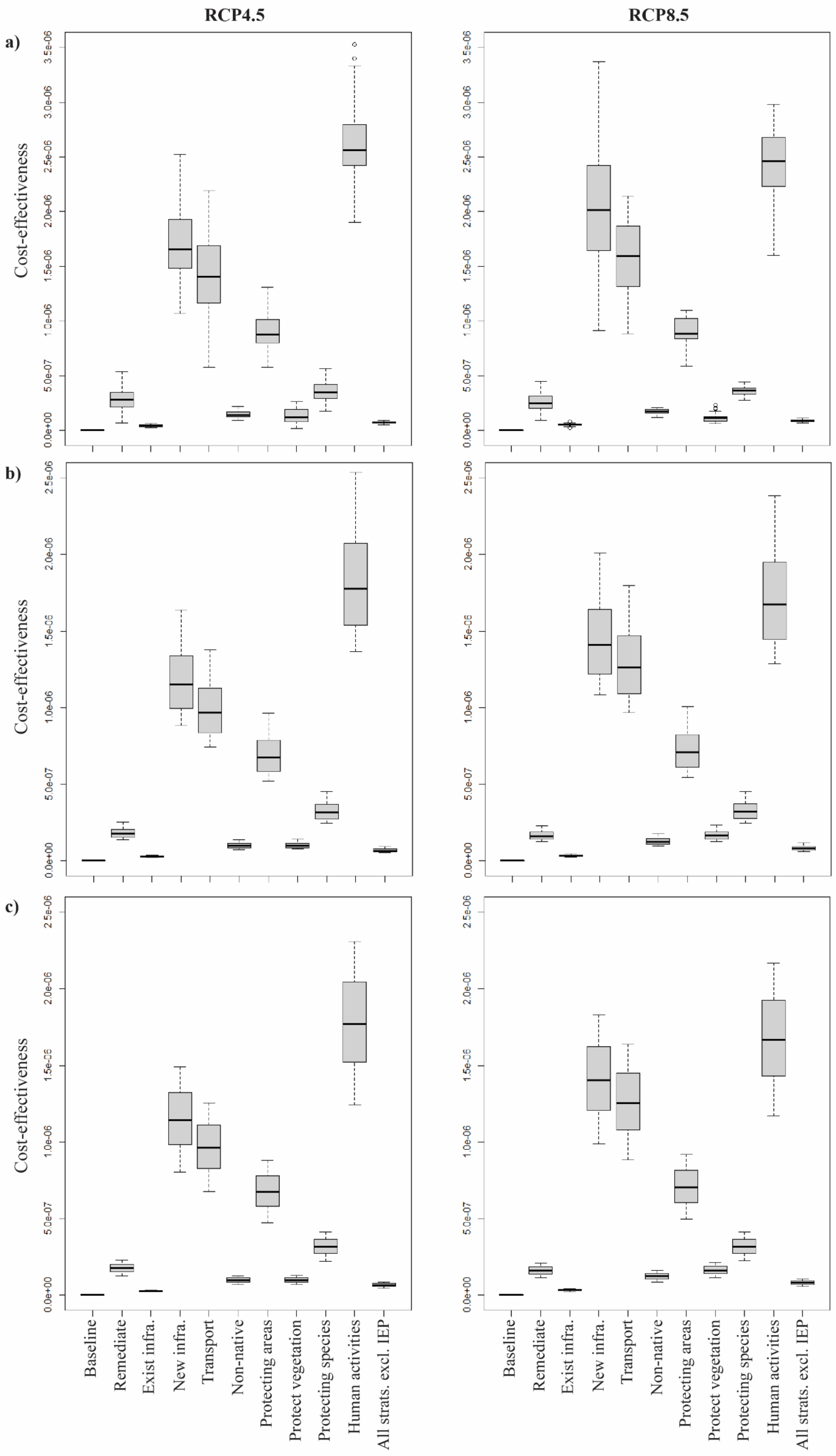

RCP4.5
RCP8.5
a)
b)
c)
Cost-effectiveness
0.0e+00
5.0e-07
1.0e-06
1.5e-06
2.0e-06
2.5e-06
3.0e-06
3.5e-06
Baseline
Remediate
Exist infra.
New infra.
Transport
Non-native
Protecting areas
Protect vegetation
Protecting species
Human activities
All strats. excl. IEP

Figure E. Output from the sensitivity analysis examining sources of expert uncertainty on the cost-effectiveness ranking of strategies under two climate forcing scenarios (RCP4.5, RCP8.5). Sources of uncertainty include: *a)* expected benefit, *b)* cost, *c)* feasibility. While each of the sources was considered independently, the other two variables/sources used the experts original best-estimates in the cost-effectiveness equation. The whiskers of the boxplots can be considered to represent the most optimistic (maximum) and most pessimistic (minimum) outcomes possible for each strategy. The data underlying this Figure can be found in S2 Data.

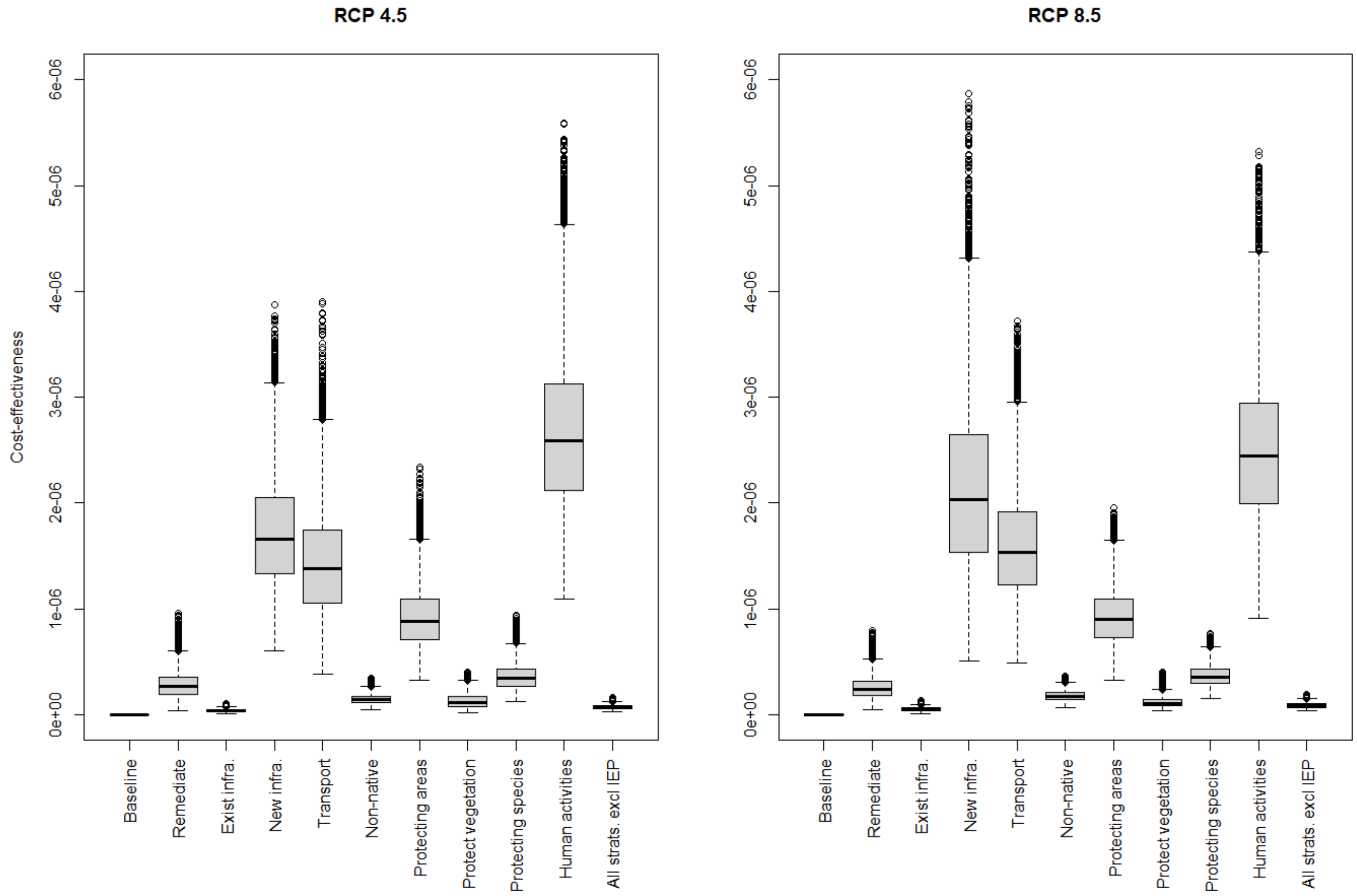

Figure F. Output from the sensitivity analysis examining sources of expert uncertainty on the cost-effectiveness ranking of strategies, whilst varying all three sources of expert uncertainty (expected benefits, cost, feasibility) together. The whiskers of the boxplots can be considered to represent the most optimistic (maximum) and most pessimistic (minimum) outcomes possible for each strategy. Outcomes under two climate forcing scenarios (RCP4.5, RCP8.5) are represented. The data underlying this Figure can be found in S2 Data.

Supplementary Tables

Table A. The Seven (8) shortfalls of biodiversity knowledge as defined by [1], with the addition of an 8th strategy (Galilean).

Shortfall	Description
Linnean	Uncertainties regarding the number of species on Earth and the fact that many species remain undescribed or uncatalogued
Wallacean	Uncertainties regarding geographic distribution of species
Prestonian	Uncertainties regarding species population dynamics and abundance
Darwinian	Uncertainties regarding relatedness and evolution of species and traits
Raunkiæran	Uncertainties regarding species functional traits and ecological functions
Hutchinsonian	Uncertainties regarding species response to abiotic factors
Eltonian	Uncertainties regarding species response to biotic factors and species interactions
Galilean	Uncertainties regarding technological processes; where we have the data available but have not yet developed or understood the technology required to interpret it

Table B. Shortfalls identified per taxon that limit understanding and assessment of 38 Antarctic taxa. Presence of shortfall indicated by a tick.

Taxon	Darwinian	Eltonian	Gallilean	Hutchinsonian	Linnean	Prestonian	Raunkioren	Wallacean
Adélie penguins	✓	✓	✓	✓	✗	✓	✗	✗
Antarctic shag	✗	✓	✗	✗	✗	✓	✗	✗
Bank-forming mosses	✗	✓	✗	✓	✓	✓	✓	✓
Biological soil crust comm.	✗	✓	✗	✓	✓	✓	✗	✓
Chinstrap penguins	✓	✓	✗	✗	✗	✓	✗	✗
Colobanthus quitensis	✗	✓	✗	✓	✗	✓	✗	✓
Crustose lichens	✓	✓	✓	✓	✓	✓	✓	✓
Deschampsia antarctica	✗	✓	✗	✓	✗	✓	✓	✓
Dry soil microbial communities	✓	✓	✓	✓	✓	✓	✓	✓
Dry soil nematodes	✗	✓	✗	✓	✗	✓	✗	✓
Emperor penguins	✓	✓	✗	✓	✗	✓	✗	✗
Entomobryomorpha springtails	✗	✓	✗	✓	✗	✓	✓	✓
Free-living mites	✗	✓	✗	✓	✓	✓	✗	✓
Freshwater nems,rotis,tardis	✓	✓	✗	✓	✓	✓	✗	✓
Freshwater or limnetic algae	✓	✓	✗	✓	✓	✓	✗	✓
Fruiticose & foliose lichens	✓	✓	✗	✗	✗	✓	✗	✓
Gentoo penguins	✗	✓	✗	✓	✗	✓	✗	✗
Greater sheathbill	✗	✓	✗	✗	✗	✓	✗	✗
Hydric mosses	✓	✓	✗	✓	✓	✓	✓	✓
Intertidal oligochaetes	✗	✓	✗	✓	✓	✓	✓	✗
Intertidal mites	✗	✓	✗	✓	✓	✓	✗	✓
Intertidal springtails	✗	✗	✗	✗	✗	✗	✗	✗
Leafy liverwort	✓	✓	✗	✓	✓	✓	✓	✓
Lithic microbial communities	✗	✗	✗	✓	✗	✗	✗	✗
Mat-forming terrestrial algae	✗	✓	✓	✓	✓	✓	✓	✓
Mesic mosses	✓	✓	✗	✓	✗	✓	✓	✓
Microbial mats (lake and flush)	✓	✓	✓	✓	✓	✓	✓	✓
Midges	✗	✓	✗	✓	✗	✓	✗	✓

Moss associated nems,rotis,tardis	✗	✓	✗	✓	✓	✓	✓	✓
Penguin rookery assoc. nematodes	✗	✗	✗	✗	✗	✗	✗	✗
Poduromorpha springtails	✗	✓	✗	✓	✗	✓	✓	✓
Procellariids	✓	✓	✗	✗	✗	✓	✗	✓
South polar skua	✗	✓	✗	✗	✗	✓	✗	✗
Southern giant petrels	✗	✓	✗	✓	✗	✓	✗	✗
Truly aquatic mosses	✓	✓	✗	✗	✗	✓	✗	✓
Wet soil microbial comm.	✓	✓	✓	✓	✓	✓	✓	✓
Wet soil nems,rotis,tardis	✓	✓	✗	✓	✓	✓	✗	✓
Xeric mosses	✓	✓	✗	✓	✓	✓	✓	✓
**TOTAL**	17	35	6	29	17	35	15	26

Table C. Average expert estimation of effort (%) required to implement conservation strategies for Antarctic terrestrial biodiversity split across two regions in Antarctica (continent and peninsula).

	**Effort split %**	
**Strategy**	**Continent**	**Peninsula**
Baseline	NA	NA
Remediation	46.7	53.3
Manage existing infrastructure	50.8	52.5
Manage new infrastructure	59.2	40.8
Transport management	36.7	63.3
Manage non-native species and disease	33.3	66.7
Protect vegetation from physical impacts	0.0	100.0
Protecting areas	46.7	53.3
Managing and protecting species	50.0	50.0
Minimise impacts of human activity	41.7	58.3
Influence external policy	50.0	50.0
All strategies excl IEP	39.3	61.1
All strategies combined	40.3	60.1

Table D. Evaluation of key management strategies for Antarctic biodiversity until 2100 under two climate forcing scenarios (RCP4.5, RCP8.5) in two regions a, continent, b, peninsula. Including estimated total cost over the next 83 years (present value; using social discount rate of 2%), estimated feasibility, total expected benefit (% change combined for all taxa), and cost-effectiveness rank. Ranked in order of cost-effectiveness (CE) for RCP4.5. Benefits of strategies are calculated using best estimates of improved intactness provided by experts. Values used to calculate benefit were capped at current (100%) intactness (An1).

a) Continent

			RCP4.5		RCP8.5	
**Strategy**	**NPV (US$ M)**	**Feasibility**	**Benefit**	**Rank**	**Benefit**	**Rank**
Modify human activities	4.78	0.31	19.2	1	15.3	2
Transport management	14.33	0.72	18.2	2	23.3	1
Manage new infrastructure	18.82	0.60	21.0	3	27.3	3
Protecting areas	46.58	0.70	26.5	4	24.5	4
Manage and protect species	107.97	0.53	37.7	5	36.0	5
Remediation	51.26	0.54	12.0	6	13.0	6
Manage non-native species and disease	254.21	0.58	27.5	7	35.3	7
All strats combined excl policy influence	755.28	0.56	68.6	8	72.1	8
Manage existing infrastructure	412.48	0.60	11.2	9	11.2	9
Baseline	0	1.00	0.0	10	0.0	10
Protect vegetation	NA	NA	NA	NA	NA	NA

b) Peninsula

			RCP4.5		RCP8.5	
**Strategy**	**NPV (US$ M)**	**Feasibility**	**Benefit**	**Rank**	**Benefit**	**Rank**
Modify human activities	6.69	0.31	46.8	1	46.8	2
Manage new infrastructure	12.99	0.60	39.5	2	46.9	1
Transport management	24.75	0.72	34.2	3	45.0	3
Protecting areas	53.24	0.70	70.2	4	76.6	4
Manage and protect species	107.97	0.53	91.9	5	94.2	5
Remediation	58.58	0.54	23.8	6	19.9	6
Manage non-native species and disease	508.42	0.58	97.4	7	123.9	8
Protect vegetation	31.72	0.37	8.3	8	13.8	7
All strats combined excl policy influence	1175.68	0.56	156.2	9	205.2	9
Manage existing infrastructure	426.00	0.60	23.7	10	29.7	10
Baseline	0	1.00	0	11	0	11

Table E. Evaluation of key management strategies for Antarctic biodiversity until 2100 under two climate forcing scenarios (RCP4.5, RCP8.5). Benefit calculated using a, upper bound values and b, lower bound values given by experts. Including estimated total cost over the next 83 years (present value; using social discount rate of 2%), estimated feasibility, total expected benefit (% change combined for all taxa), and cost-effectiveness rank. Ranked in order of cost-effectiveness (CE) for RCP4.5. Values used to calculate benefit were capped at current (100%) intactness (An1).

**a) Upper bound**			**RCP4.5**		**RCP8.5**	
**Strategy**	**NPV (US$ M)**	**Feasibility**	**Benefit**	**Rank**	**Benefit**	**Rank**
Minimise impact of human activity	11.48	0.31	38.5	1	45.1	2
Transport management	39.08	0.72	42.8	2	49.1	3
Manage new infrastructure	31.81	0.60	35.8	3	65.5	1
Protecting areas	99.82	0.70	68.2	4	77.2	4
Manage and protect species	215.94	0.53	89.2	5	111.2	5
Remediation	109.85	0.54	36.4	6	27.5	7
Protect vegetation	31.72	0.37	6.8	7	14.2	6
Manage non-native species and disease	762.62	0.58	67.0	8	118.9	9
All strategies combined	1937.97	0.51	162.3	9	369.3	8
Manage existing infrastructure	811.43	0.60	20.3	10	30.5	10
Baseline	0	1.00	0	11	0	11

**b) Lower bound**			**RCP4.5**		**RCP8.5**	
**Strategy**	**NPV (US$ M)**	**Feasibility**	**Benefit**	**Rank**	**Benefit**	**Rank**
Minimise impact of human activity	11.48	0.31	154.8	1	138.2	1
Manage new infrastructure	31.81	0.60	163.3	2	170.6	2
Transport management	39.08	0.72	135.0	3	137.1	3
Protecting areas	99.82	0.70	231.5	4	219.5	4
Manage and protect species	215.94	0.53	214.5	5	211.0	5
Remediation	109.85	0.54	102.0	6	88.0	6
Manage non-native species and disease	762.62	0.58	441.2	7	476.9	7
Protect vegetation	31.72	0.37	20.9	8	12.4	8
All strats combined excl policy influence	1923.84	0.56	374.1	9	390.9	9
Manage existing infrastructure	811.43	0.60	117.0	10	113.4	10
Baseline	0	1.00	0	11	0	11

Table F. The three most cost-effective conservation management strategies for Antarctic taxa under climate forcing scenario RCP8.5; identified in order of significance as 1, 2, 3. Benefits of strategies are calculated using best estimates of improved intactness provided by experts and were capped at current (100%) intactness (An1; if a taxon only has benefits beyond 100% no strategies have been identified; see Table H including these benefits). The cost-effectiveness values were calculated using a discount rate of 2% for the present value.

Taxon	Remediate	Exist Infra	New Infra	Transport	Non-native	Protect Vegetation	Protecting Areas	Protecting Species	Human Activities
Adelie Penguins			3	1					2
Antarctic shag			3	2					1
Bank-forming Mosses*									
Biological soil crust communities							2		1
Chinstrap Penguins			3			2	1		
Colobanthus quitensis*									
Crustose lichens*									
Deschampsia antarctica*									
Dry soil microbial communities*									
Emperor Penguins				1	3			2	
Entomobryomorpha springtails			2				3		1
Freshwater nems,rotis,tardis							1		
Freshwater or Limnetic algae*									
Fruiticose & Foliose lichens*									
Gentoo Penguins						3	2		1
Greater sheathbill				1			3	2	
Hydric Mosses*									
Intertidal oligochaetes					1				
Intertidal mites*									
Intertidal springtails*									
Leafy Liverwort			1	2					3
Lithic microbial communities*									
Mat-forming terrestrial algae*									
Mesic Mosses*									
Microbial mats*									
Midges		3	1		2				
Poduromorpha springtails			2	3					1
Procellariids			2	1			3		
South Polar Skuas									
Southern giant petrels			2	3					1
Terrestrial, dry soil nematodes			1				3		2
Terrestrial, free-living mites									
Moss associated nems,rotis,tardis			1				3		2
Penguin rookery associated nematodes								2	1
Wet soil nems,rotis,tardis					1				
Truly aquatic mosses*									
Wet soil microbial communities*									
Xeric Mosses			1	2					3

*this taxon not expected to benefit from any strategy

Table G. The three most cost-effective conservation management strategies for Antarctic taxa under climate forcing scenario RCP4.5; identified in order of significance as 1, 2, 3. Benefits of strategies are calculated using best estimates of improved intactness provided by experts and were capped at current (100%) intactness (An1; if a taxon only has benefits beyond 100% no strategies have been identified; see Table I including these benefits). The cost-effectiveness values were calculated using a discount rate of 2% for the present value.

Taxon	Remediate	Exist Infra	New Infra	Transport	Non-native	Protect Vegetation	Protecting Areas	Protecting Species	Human Activities
Adelie Penguins				1			3		2
Antarctic shag			3	2					1
Bank-forming Mosses			2	3					1
Biological soil crust communities							2	3	1
Chinstrap Penguins			3			2	1		
Colobanthus quitensis*									
Crustose lichens*									
Deschampsia antarctica*									
Dry soil microbial communities*									
Emperor Penguins				1	3			2	
Entomobryomorpha springtails			2				3		1
Freshwater nems,rotis,tardis							1		
Freshwater or Limnetic algae			2				3		1
Fruiticose & Foliose lichens*									
Gentoo Penguins						1	2		3
Greater sheathbill				1			2	3	
Hydric Mosses*									
Intertidal oligochaetes*									
Intertidal mites*									
Intertidal springtails*									
Leafy Liverwort			1	2			3		
Lithic microbial communities*									
Mat-forming terrestrial algae*									
Mesic Mosses*									
Microbial mats*									
Midges			1		3		2		
Poduromorpha springtails			2	3					1
Procellariids	1		1	2					
South Polar Skuas*									
Southern giant petrels			2	3					1
Terrestrial, dry soil nematodes			1				3		2
Terrestrial, free-living mites*									
Moss associated nems,rotis,tardis			1					3	2
Penguin rookery associated nematodes								2	1
Wet soil nems,rotis,tardis					2			1	
Truly aquatic mosses*									
Wet soil microbial communities*									
Xeric Mosses			1	2			3		

*this taxon not expected to benefit from any strategy

Table H. The three most cost-effective conservation management strategies for Antarctic taxa under climate forcing scenario RCP8.5; identified in order of significance as 1, 2, 3. Benefits of strategies are calculated using best estimates of improved intactness provided by experts and include benefits to taxa expected to expand beyond current (100%) intactness (An2). The cost-effectiveness values were calculated using a discount rate of 2% for the present value.

Taxon	Remediate	Exist Infra	New Infra	Transport	Non-native	Protect Vegetation	Protecting Areas	Protecting Species	Human Activiti
Adelie Penguins				1			3		2
Antarctic shag			3	2					1
Bank-forming Mosses				3		2			1
Biological soil crust communities							2	3	1
Chinstrap Penguins						1	2	3	
Colobanthus quitensis			3			2			1
Crustose lichens			3				1		2
Deschampsia antarctica			3			2			1
Dry soil microbial communities				1			3		2
Emperor Penguins				1	3			2	
Entomobryomorpha springtails			2	3					1
Freshwater nems,rotis,tardis			3	2					1
Freshwater or Limnetic algae			3	2					1
Fruiticose & Foliose lichens			2	3					1
Gentoo Penguins			1			3			2
Greater sheathbill				1			3	2	
Hydric Mosses			2	3					1
Intertidal oligochaetes					1				
Intertidal mites*									
Intertidal springtails*									
Leafy Liverwort			2	3					1
Lithic microbial communities*									
Mat-forming terrestrial algae	1								
Mesic Mosses			1	3					2
Microbial mats					2		1		
Midges			2	3					1
Poduromorpha springtails			2	3					1
Procellariids	3		2	1					
South Polar Skuas*									
Southern giant petrels			3	2					1
Terrestrial, dry soil nematodes			1				3		2
Terrestrial, free-living mites			1			2			
Moss associated nems,rotis,tardis			1	3					2
Penguin rookery associated nematodes								2	1
Wet soil nems,rotis,tardis			3	2					1
Truly aquatic mosses			2	3					1
Wet soil microbial communities			3				2		1
Xeric Mosses			2	3					1

*this taxon not expected to benefit from any strategy

Table I. The three most cost-effective conservation management strategies for Antarctic taxa under climate forcing scenario RCP4.5; identified in order of significance as 1, 2, 3. Benefits of strategies are calculated using best estimates of improved intactness provided by experts and include benefits to taxa expected to expand beyond current (100%) intactness (An2). The cost-effectiveness values were calculated using a discount rate of 2% for the present value.

Taxon	Remediate	Exist Infra	New Infra	Transport	Non-native	Protect Vegetation	Protecting Areas	Protecting Species	Human Activi
Adelie Penguins				1			3		2
Antarctic shag			3	2					1
Bank-forming Mosses				3		1			2
Biological soil crust communities							2	3	1
Chinstrap Penguins						3	1	2	
Colobanthus quitensis			2	3					1
Crustose lichens			3				1		2
Deschampsia antarctica			2			3			1
Dry soil microbial communities				1			3		2
Emperor Penguins				1	3			2	
Entomobryomorpha springtails			2	3					1
Freshwater nems,rotis,tardis			3	2					1
Freshwater or Limnetic algae			2	3					1
Fruiticose & Foliose lichens			2	3					1
Gentoo Penguins			2			3	1		
Greater sheathbill				1			3	2	
Hydric Mosses			2	3					1
Intertidal oligochaetes*									
Intertidal mites*									
Intertidal springtails*									
Leafy Liverwort			3	2					1
Lithic microbial communities*									
Mat-forming terrestrial algae	2				3				1
Mesic Mosses			1	3					2
Microbial mats			2				3		1
Midges			2	3					1
Poduromorpha springtails			2	3					1
Procellariids	3		1	2					
South Polar Skuas*									
Southern giant petrels			2	3					1
Terrestrial, dry soil nematodes			1				3		2
Terrestrial, free-living mites			2			1			
Moss associated nems,rotis,tardis			1	3					2
Penguin rookery associated nematodes								2	1
Wet soil nems,rotis,tardis			3	2					1
Truly aquatic mosses			2	3					1
Wet soil microbial communities			2			3			1
Xeric Mosses			2	3					1

*this taxon not expected to benefit from any strategy

Table J. Methodological terms relating to priority threat management.

Term	Definition
Taxon/Feature	The individual species or group of species that have been grouped together by the biodiversity experts. Each taxon represents a group of species that are predicted to respond similarly to threats and management in the future. See table S2 for a list of included taxa.
Threats	The threats identified by the experts that impact or are likely to impact Antarctic terrestrial biodiversity. See table S3 for a list of identified threats.
Region	Spatial areas defined by the experts that represent substantially different climates, biodiversity, or operations. See fig. S1 for identified regions.
Time frame	The time period over which the study occurs, in this case, the 83 years from 2017 to 2100.
Strategy	Management strategy that will provide substantial benefit to terrestrial biodiversity independently of other strategies. Each strategy has an quantifiable objective and consists of a set of actions that when implemented will achieve the objective. See Table 1 for a list of included strategies and table S4 for a detailed description of the strategies including actions, estimated cost and feasibility.
Action	Individual management activities or processes identified by experts, that will contribute to achieving overarching strategy objectives when combined. Some actions may be utilised in multiple strategies.
Baseline	A 'business as usual' strategy denoting the scenario if no new conservation strategies were to be implemented in Antarctica in the timeframe. The baseline is used to measure the benefit of each strategy. See figs. S2 and S3 for expert estimations of the baseline for each feature.
Feasibility	The predicted probability that a strategy will be successful (achieve its objectives) Calculated as a product of likelihood of uptake and likelihood of success.
Likelihood of uptake	Calculated for each individual action – the likelihood that decision makers will agree to implement the action as it is described (assuming funding is available). See table S4 for expert estimated likelihoods.
Likelihood of success	Calculated for each strategy – the likelihood that the strategy objective will be achieved when all actions have been successfully implemented. See table S4 for expert estimated likelihoods.
Cost	Calculated for each individual action – the cost of implementing the stated action. Costs were broken down into categories including salaries, berths, field and lab costs and other costs. Costs of actions are summed to calculate cost for each strategy. See table S4 for expert estimated costs.
Intactness values	The values estimated by biodiversity experts of how intact a feature is likely to be in the absence of implementation of any strategy (baseline) and how intact they are likely to be if a strategy was implemented. Intactness was estimated on a scale of 0 to 200, where a value of 100 represented no change to current (2017) intactness of that feature. Intactness can be visualised in different ways for different features, using for example, extinction risk, range contractions/expansions, density, population numbers etc. See figs. S2 and S3 for estimates of intactness values for each feature.
Best estimate	Experts best estimate of the true value of the taxon's intactness value
Lower bound	Experts best estimate of a worst-case scenario of the taxon's intactness value
Upper bound	Experts best estimate of a best-case scenario of the taxon's intactness value
Benefit	The predicted benefit that a strategy will provide to an individual feature when implemented. Benefit is calculated as the features predicted intactness values when implementing the strategy minus the intactness values estimated when implementing the baseline.<br>See figs. S7 and S8 for calculated benefit for each feature and each strategy.
Cost-effectiveness	Cost-effectiveness gives a measure of value-for-money for each strategy. It is calculated as the benefit multiplied by the feasibility and divided by the cost for each strategy and feature.

Complementarity	Complementarity analysis identifies sets of complementary strategies that when employed together will bring the maximum number of taxa possible to a specified intactness threshold (e.g. 80%) under any given budget.

Table K. The 38 taxa used in Antarctic priority threat management; where species were grouped based on a similar predicted response to threats and management strategies.

Taxon	*Feature*	*Details*
Invertebrates	Midges	The two native Antarctic midges, i.e. *Belgica antarctica, Parochlus steinenii*
	Freshwater nematodes, rotifers, tardigrades	Microfauna that survive in freshwater, e.g., nematodes - *Plectus spp*; rotifers - *Adineta grandis, Epiphanes senta*; tardigrades - *Acutuncus antarcticus*
	Terrestrial, wet soil nematodes, rotifers, tardigrades	Microfauna that primarily survive in wet soil, e.g., nematodes - *Plectus spp., Eudorylaimus antarcticus*,rotifers - *Adineta gracilis*; tardigrades
	Terrestrial, moss associated nematodes, rotifers, tardigrades	Microfauna that are associated with mosses, e.g., nematodes - *Plectus spp., Eudorylaimus*; rotifers - *Adineta grandis*; tardigrades
	Entomobryomorpha springtails	e.g. *Isotoma klovstadi*
	Poduromorpha springtails	e.g. *Gomphiocephalus hodgsoni*
	Intertidal springtails	
	Intertidal mites	
	Terrestrial, free-living mites	All free-living mites, Cryptostigmata, Prostigmata, and Mesostigmata mites. Such as *Alaskozetes antarcticus, Coccorhagidia gressitti, Gamasellus racovitzai*
	Intertidal oligochaetes	Including enchytraeids. e.g. *Christensenia spp.*
	Terrestrial, dry soil nematodes	Nematodes that primarily survive in dry soil e.g., *Scottnema lindsayae*
	Terrestrial, penguin rookery associated nematodes	Nematodes that are primarily live in association with penguin rookeries, e.g., *Panagrolaimus spp.*
Vegetation	*Colobanthus quitensis*	
	Deschampsia antarctica	
	Bank-forming Mosses	e.g. *Chorisodontium aciphyllum, Polytrichum strictum*
	Hydric Mosses	e.g. *Distichium inclinatum*
	Mesic Mosses	e.g. *Schistidium antarctici* and *Bryum pseudotriquetrum* in East Antarctica
	Xeric Mosses	e.g. *Ceratodon purpureus* in East Antarctica
	Truly aquatic mosses	i.e. lake-dwelling moss
	Leafy Liverwort	e.g. *Cephaloziella varians*
	Freshwater or Limnetic algae	
	Fruiticose & Foliose Lichens	e.g. *Usnea Antarctica, Umbilicaria*
	Crustose Lichens	e.g. *Lecanora expectans, Xanthoria elegans*
Microbes	Mat-forming Terrestrial algae	e.g. *Prasiola crispa*

	Biological soil crust communities	
	Dry soil microbial communities	Microbes that predominantly survive in the dry soil
	Lithic (endolithic, hypolithic and chasmolithic) microbial communities	Microbes that predominantly survive on/in rocks
	Microbial mats, both lake and flush systems	Cyanobacteria
	Wet soil microbial communities	Microbes that predominantly survive in the wet soil
Vertebrates (Including monospecific ectoparasites, such as lice *Lepidophthirus macrorhini*)	Adélie Penguins	
	Chinstrap Penguins	
	Emperor Penguins	
	Gentoo Penguins	
	Antarctic shag	
	Greater sheathbill	
	Southern giant petrels	
	South polar skua	
	Procellariids	Snow petrel, Southern Fulmar, Wilson's storm petrel, Antarctic prion, Antarctic petrel, Cape petrel. Includes multi-specific ectoparasties

Table L. Identified threats that may impact Antarctic terrestrial biodiversity by 2100.

Category	Threat (Activity)	Examples (Aspect)
Human Activity	Infrastructure expansion (stations, runways)	Competition with biodiversity for ice-free areas, disturbance, footpaths & physical impact, waste
	Intercontinental vehicles (planes, ships – including cargo landings)	Transfer of non-native species to the continent, pollution & oil spills, noise disturbance
	Intracontinental vehicles (ships, planes, choppers, hagglunds, etc)	Intraregional transfer of native & non-native species, pollution, disturbance (physical & noise)
	Scientific fieldwork	Physical impacts, oversampling of rare populations, noise disturbance (eg. drones), non-recovery of scientific equipment
	Tourist landings	Transfer of non-native species to the continent, intraregional transfer of native & non-native species, pollution & oil spills, disturbance (physical and noise)
	Fisheries	Impact on terrestrial food webs through overfishing
Non-native species	Introduction of non-native flora	Competition, reduced diversity, extinctions of native species
	Introduction of non-native fauna	Competition, reduced diversity, extinctions of native species
	Established non-native flora	Competition, reduced diversity, extinctions of native species
	Established non-native fauna	Competition, reduced diversity, extinctions of native species
	Introduced disease or virus	Non-native microbes, native population impacts and declines, extinctions
Native species	Fur seal expansion	Physical impacts on native species, range contractions, localised extinctions. All impacts may be exacerbated if climate change further facilitates fur seal expansion
Climate Change (Direct)	Increasing temperatures	Increasing or decreasing performance of native species, range expansions and contractions, competition, extinctions
	Changing precipitation	Increasing or decreasing performance of native species, range expansions and contractions, competition, extinctions
Climate Change (Indirect)	Changing sea ice	Vertebrate population contractions or expansions
	Changing sea-level	Range contractions or expansions, extinctions
	Expansion of ice-free areas	Increased habitat and connectivity – increasing gene flow, population expansion, increasing competition, movement of non-native species
	Ocean acidification	Impact on terrestrial species through impacts on marine food web
	Ozone hole recovery	Changes in UV radiation levels
	Changing wind patters	Further impacts on local climate
	Native species expansion	Competition for resources
Pollution (Long range)	Macroplastic	
	Microplastics	Impact on terrestrial species through marine food web
	POP’s (Persistent Organic Pollutants)	Decreasing performance
Pollution (Local)	Stations (current)	Human waste, contamination, CO2 emissions
	Vehicles	Oil spills, CO2 emissions
	Legacy waste	Persisting contamination

Table M. Estimates for common priority threat management costs averaged from various National Antarctic Program estimates of cost; see S1 Data for further details.

**Item**	**Average cost (US$)**
Office FTE	55,224
Antarctic FTE	91,988
Short summer berth	56,871
Long summer berth	102,846
Winter berth	288,905
Intra-regional flight	16,335
Inter-regional flight	96,042
Laboratory costs	35,714
Workshop	71,429
Satellite images (per site)	714
Captive breeding program	1,750,000

Table N. Percentage of Antarctic biodiversity taxa likely to decline, remain neutral, or benefit with future changes regionally and overall by 2100, under two analyses: **a,** when intactness values were capped at current (100%) intactness (An1), and **b,** when intactness values include benefits to taxa expected to expand beyond current (100%) intactness (An2). The values given represent biodiversity experts best estimate of the true intactness value. Estimates given under two climate forcing scenarios: RCP4.5 and RCP8.5.

a) An1 - Excluding benefits >100% intactness

% taxa that decline			
**RCP**	**Continent**	**Peninsula**	**Overall**
4.5	57.1	62.2	65.8
8.5	64.3	62.2	63.2
% taxa that are neutral			
**RCP**	**Continent**	**Peninsula**	**Overall**
4.5	42.9	37.8	34.2
8.5	35.7	37.8	36.8
% taxa that benefit			
**RCP**	**Continent**	**Peninsula**	**Overall**
4.5	NA	NA	NA
8.5	NA	NA	NA

b) An2 - Including benefits >100% intactness

% taxa that decline			
**RCP**	**Continent**	**Peninsula**	**Overall**
4.5	35.7	40.5	44.7
8.5	35.7	37.8	39.5
% taxa that are neutral			
**RCP**	**Continent**	**Peninsula**	**Overall**
4.5	17.9	5.4	5.3
8.5	10.7	8.1	7.9
% taxa that benefit			
**RCP**	**Continent**	**Peninsula**	**Overall**
4.5	46.4	54.1	50
8.5	53.6	54.1	52.6

References

1. Hortal J, Bello Fd, Diniz-Filho JAF, Lewinsohn TM, Lobo JM, Ladle RJ. Seven shortfalls that beset large-scale knowledge of biodiversity. Annual Review of Ecology, Evolution, and Systematics. 2015; 46(1):523-49.
