# Supplementary material for: Threat management priorities for conserving Antarctic biodiversity
Source: PLoS Biol. 2022 Dec 22;20(12):e3001921. doi: 10.1371/journal.pbio.3001921 (PMC9778584; doi:10.1371/journal.pbio.3001921)
Supplement: Attachment — Submitted filename: Lee_PTM_Response-to-reviewers.pdf [file pbio.3001921.s005.pdf]

Editors Comments:

IMPORTANT: You'll see that there is a strong split in opinion among the reviewers. Reviewer #3 is very positive, and reviewer #1 somewhat so. However, rev #1 wants you to re-structure the paper and give substantially more methodological detail. Reviewer #2 recommended that we Reject your paper, raising three major concerns. After some discussion with the Academic Editor, we'd like to give you an opportunity to review, but I shall transmit the following [somewhat edited] comments from the Academic Editor to give you an idea of how we think you should address this reviewer's concerns: "Reviewer #2 also seems to echo my original concerns by accepting that expert guesses are necessary but asking how good are these "guesses"?... One could ask how often experts actually get it right?... I am trying to be somewhat provocative, but this question from reviewer #2 does seem like the linchpin of any attempt to revise the paper... I think the paper will ultimately be substantially improved by challenging the authors to address the comments from #2 with actual revisions to the text and analyses."

We found the reviewers comments constructive and thoughtful, and we believe that addressing them has helped to greatly improve the manuscript. In response to Reviewer #1's comments we have expanded and clarified the methods summary section in the main text, as well as clarified other details throughout as suggested (see the point-by-point responses below). We appreciate both the Academic Editor and Reviewer #2's concerns around the uncertainty of expert estimates in conservation assessments. During the expert elicitation we did collect measures of uncertainty, including a range of estimates for species intactness (lower and upper bounds, and a best estimate) and a confidence measure. Cost-effectiveness estimates using the upper and lower bounds were incorporated into the supplementary material, though maybe not given the attention they deserved in the original text. We have now carried out a formal sensitivity analysis of the uncertainty in the cost-effectiveness rankings using Monte Carlo simulations, and also included an additional section dedicated to uncertainty in the Discussion. We found that the most cost-effective strategies (minimising impacts of human activities, managing new infrastructure and transport management) were consistently identified as the most cost-effective regardless of all sources of uncertainty. We also appreciate Reviewer #3's enthusiasm for our study and believe this work will be of broad interest.

Reviewers Comments:

Reviewer #1:

The conservation of Antarctic biodiversity is clearly of international interest and a subject that needs more to be written about it. I applaud the basic work done and the intention behind it. However, I am afraid that the manuscript, as written, is not yet of sufficient clarity to allow an adequate review. I have attempted to make a few comments along the way that might assist, and some of them will simply show the level of confusion engendered in the reader at present. But in general, can I suggest that the authors engage an experienced communicator to give more shape to what could clearly be an important study?

We thank the reviewer for highlighting areas where more clarity was needed - we have addressed the reviewers' specific comments (see below), and have also gone through the text carefully and reworded a number of sentences to ensure their meaning and/or intent is clear.

Introduction: Conservation ROI is controversial because of opposition to the idea "cheapest is best". At present, the introduction limits itself to posing the ROI question without set-up motivation for why it's worth asking. Given the controversy, I believe it's important for the authors to set up the motivation and acknowledge why the two questions being asked are important, and are different in their interest.

We agree that this is a topic worthy of introduction that we initially missed. We have now included an introductory section dedicated to cost-effectiveness approaches (Lines 122 – 128).

Line 103: The authors write: "We apply a structured, participatory decision-science approach 104 (Carwardine et al. 2012, 2019) to quantify and prioritise cost-effective and complementary 105 strategies for achieving biodiversity conservation in terrestrial Antarctica (see Materials and 106 Methods). This approach is especially suited to the Antarctic as the continent's isolation, 107 extreme climate and the relatively late initiation of scientific activities (which accelerated 108 following the International Geophysical Year of 1957-1958; Summerhayes 2008) have resulted 109 in a lack of quantitative data, especially on species distributions, interactions and taxonomy".

This phrase suggests that the method was chosen a priori as some sort of ideal. There is a little bit of overenthusiastic marketing going on, I feel. There is nothing wrong with saying that the continent is data-poor and so one has to rely on expert-based approaches, acknowledging very briefly the strengths and limitations of such approaches. Line 110 can then be a context-specific defence of the approach for this study. As the sentence stands, it unfortunately implies special pleading, and many readers will actually lose confidence in what is to follow. It's the rhetorical difference between stating your approach and honestly defending it (with acknowledgment of any limitations), versus claiming that you chose the cutting-edge method as a claim to credibility.

We have reworded and reorganised the description of the expert approach as suggested (Lines 149-158).

Additionally, please start a new section that begins with whatever replaces this sentence. As it stands, it is not clear that the summary-of-methods has begun at this point. It would be much clearer if it were subtitled or otherwise better signposted.

We thank the reviewer for this suggestion, we have done this here (Line 147) and throughout in response to Rev #3's comment on headings.

Line 115: I suggest this also needs to be signposted - "for our survey" would do - and flow from the defence of expert-based methods. Also, the very short paragraph feels chopped up.

We have added a signpost "First" (Line 160), carefully revised the structure of this paragraph and expanded a number of details in response to other comments, all of which have improved the flow.

Line 120: if the methods are based on benefit and cost, it feels incomplete to summarize only the benefit method. A sentence here about the cost approach would be best advised.

We have added a couple of sentences about cost in Lines 169-175.

Line 130: this entire paragraph is confusing because it starts by saying there are two RCPs (4.5 and 8.5) and then immediately talks about RCP2.6 instead. Do the authors mean to say that they assessed the option of influencing climate policy as one threat abatement measure in isolation, and then asked the experts about the likely outcomes from a basket of non-policy-based, more local abatement measures under two different climate scenarios?

That is what we meant to say and we agree this was confusing. We have reworded the text in Lines 197-207.

Line 148: please signpost with something like "In our results, we found that" to make it clearer what is happening here.

We have added a heading in Line 215.

Also, if there is going to be a results section on threat, then surely it is important to set up a study of threat in the questions asked and in the brief methods description? An alternative - and perhaps a better one - is to move this entire section into the online material. As it stands, it is a sudden rush of highly detailed, descriptive work that seems to confuse the reader about where the ms is going. Case studies are useful but when the paper is a high-level piece, it becomes difficult to follow if suddenly sidetracked into a long summary of case-specific details.

We agree this needed setting up in the introduction and have now done so (Lines 110-120). We believe the vulnerability results are an important output and should be kept in the main text, thus we have also added this as a third research question (Line 138).

Lines 157-9: again, this seems to mix up sections of the logic flow. I would suggest putting the threat to Emporers as an important point of interest for the lay reader, near the top of the introduction, and then the assumed outcome of climate change mitigation on Emporers in a later section where the "influence policy" option is being set up/motivated.

With the additional information in the Introduction now better setting up the threat vulnerability results, we think that it is appropriate to keep the Emperor vulnerability section where it is. In our view it nicely highlights the alignment between what we found and what the literature shows.

Line 179: These details might be better off in a caveats section that is either in the online material, or more towards the end of the ms. It would help the reader to have a quicker transition from question->method->result->discussion, implication and caveat.

We think this is a good suggestion and have now moved the shortfalls discussion to a new discussion section on uncertainty, where we discuss the sensitivity analysis and sources of uncertainty (Lines 505-527).

Line 194 and figure 1: Please define peninsula/continent and explain its importance before mentioning it here.

We have added a couple of sentences defining the peninsula/continental regions and why it is important to consider them separately in Lines 162-164.

The figure is important but highly confusing: it is not immediately apparent that part (a) continues down the page. The message that some taxa will do worse and some will do better is a major result but has got lost graphically, and was not clearly and simply presented in the text either.

We have tried to simplify the figure by reducing the information in b) and c) to make it clearer that a) continues down the page. We have added a sentence in Lines 220-222 highlighting the important point that some taxa will do worse and some better.

It would help greatly if these points were clarified, please. Also, there seems to be a graphical mix-up between response to all threats and response to climate change alone. I'm afraid it's entirely confusing and leaves the reader wondering what results are being presented at a fundamental level.

We can see why this is confusing, the vulnerability is vulnerability to all threats, though we assume that taxa that benefit in the future are benefitting from climate change (rather than from the impact of other threats). We have simplified b) to a simple map of the two regions and removed reference to climate change responses, which we believe will reduce reader confusion. We have also reworded the legend of part a) from 'Climate change status' to 'Response to threats' which we believe will also reduce confusion.

Part (b) is similarly lacking in a legend or explanation: is this a spatially explicit mapping of threat levels and if so, where is the key? And in the caption, how are the expected changes in outcome from different levels of intervention being included and measured? This seems to be suddenly implied as a method with no explanation leading up to it.

As the assessment was carried out for both regions separately (with regional benefits being summed together for overall benefits) part b) was summarising some of these regional differences. We agree it was overly complicated though and have simplified part b) to a simple map of the two regions.

Line 222: I am again confused. Firstly, are the authors really saying that climate change is by far the biggest threat, but achieving the Paris commitments will make the same difference whether we assume large or small forcing? That is extremely counterintuitive and demands explanation and defending (including a clearer sense of the timelines involved in threat abatement and species decline). More generally, I find it very hard to decipher what this paragraph is trying to present.

We have reworded this sentence to make the message clearer (Lines 290-291), which is that the ranking of the strategies stays the same regardless of the climate scenario (not that the amount of benefit is the same for each).

Line 232: what is the meaning of the phrase "Whilst most strategies provide only benefits to taxa"? I assume the intention is to say that most interventions are expected to reduce decline but some could actually have perverse outcomes and increase decline for some taxa?

We have also reworded these couple of sentences to clarify that it is not that we expect perverse outcome from some strategies, but that reducing the climate forcing scenario (e.g. from RCP4.5 to RCP2.6) could lead to less benefit for some of those species that are expected to benefit from climate change (Lines 302-306).

Line 302: the authors now state what was already a red flag - that it would be almost impossible to put a cost on "influencing global climate policy". It would also be nearly impossible to measure the outcome per dollar. It is therefore surprising that the authors only acknowledge that problem at this late stage and omit policy influencing from ROI assessment, and also that they see no difficulty in comparing the costs (including those for policy influencing) at other points in the ms.

We agree this is a really important point and that it should have been made clearer earlier in the manuscript. We have thus highlighted this information at appropriate points in the methods summary of the introduction (Lines 164-167, 175-181). Whilst we mention the small costs of the *'Influence external policy'* strategy in the 'feasibility and costs' section we did not include this strategy in the cost-effectiveness or complementarity analyses (except in one

case in S5 Fig. where we examine whether more taxa are able to reach complementarity intactness thresholds if climate change could be reduced to RCP2.6). We have also removed the discussion around climate mitigation costs in response to this comment and that of Reviewer #2.

Line 333: the key figure here is surely that the $1.9 billion is spread over most of the century and equates to $23 million per year. This is not what the average reader is going to take away from the abstract - including a policymaker - when the abstract only mentions the $1.9 billion. Indeed, it makes little sense to state a total budget that is expected to magically end in exactly 83 years time. Why not lead on the annual cost, which is far more compelling? Similarly, in lines 361-374, annualizing all values would make much more policy sense.

We initially used total cost as the costs are not borne evenly across years (it requires a higher investment in the earlier years than later years). However, we agree that annual cost is more useful and have converted to annual costs in both abstract (Line 67) and discussion (Lines 376-379).

Reviewer #2:

The paper describes the results of a solicitation of expert opinion to assess priorities for threat management to conserve Antarctic biodiversity. The paper is written clearly and well-executed. The authors provide a very clear statement of the questions to be addressed (lines 97-98). I think the authors have done about as well as could be done given the limited set of information they have to work with. The editorial question is whether this is enough to justify publication in PLOS Biology.

The main weakness of the paper is the lack of underlying data to support the kind of cost-effectiveness exercise the authors want to carry out. We just don't know that much about Antarctic biodiversity. There is very little hard evidence on which to base an assessment. How would various taxonomic groups respond to various conservation strategies (what are the benefits)? What are the costs of various strategies? What is the likely feasibility and the likelihood of success? The lack of hard evidence means we have to rely on expert judgement. The experts probably have as good of a guess as anyone about these questions, but how good these guesses are, is itself a guess.

We appreciate the concerns of both the reviewer and the editor regarding the uncertainty of expert judgement. We have undertaken a thorough sensitivity analysis as suggested below to test the robustness of the cost-effectiveness rankings in light of the three major sources of expert uncertainty (estimates of benefit, cost and feasibility). The sensitivity analysis is detailed in Lines 959-977 and we have also added a new section to the Discussion dedicated to uncertainty (Lines 505-527). The results of the sensitivity analysis (Supplementary Figures S6 and S7) indicate that the same strategies are consistently identified as the most cost-effective regardless of the sources of uncertainty.

A second weakness, which again is largely outside the control of the authors, is that the main threat to Antarctic biodiversity seems to be climate change, which means that reducing threats lies well beyond the Antarctic. Climate change clearly poses a threat to some species. However, if it is really the case that "Some taxa do not appear to be sensitive to climate change and their intactness is expected to remain similar to current levels" (lines 168-169), then this makes it seem as if climate change it the overriding threat and little else matters, though later the authors indicate that other strategies benefit some taxa.

This is one of the major results of the study – that climate change is the overriding threat to Antarctic biodiversity and that there is more to be done by the Antarctic community in this space (who have tended to focus on local actions in the past, such as non-native species management). However, our results clearly show the benefits of more local actions, and we believe it is also important to continue highlighting these actions, which can be implemented relatively easily and are invariably more feasible. The cost-effectiveness analysis identified several of these local actions that are likely to be extremely cost-effective, but which have been given little consideration by Antarctic Treaty Parties and National Antarctic Programmes to date. We have tried to ensure this is now clear throughout the manuscript.

On climate change, there is little that the authors of this paper can add to the large literature on costs and feasibility of various climate policies and pathways and I'm not convinced that they should devote space to talking about the larger costs or benefits of addressing climate change.

We initially considered it important to discuss the wider benefits of addressing climate change, though agree we cannot add to the literature on this and have removed this discussion as suggested. We still think it is important that every region or sector contributes to global solutions though and have hence kept the cost information about the *'Influence global policy'* strategy to highlight that there are still things the Antarctic Treaty can contribute to global climate discussions.

A third weakness, but one on which the authors could do something more to address, is to convince those outside the Antarctic research community that conservationists should be paying greater attention to Antarctica. Because Antarctica is relatively species poor and "is relatively free of many of the environmental threats that beset the rest of the world" (lines 76-77), Antarctic conservation doesn't seem likely to rank highly in terms of global conservation priorities. If this is incorrect, the authors should provide some evidence or reasoning why Antarctic conservation should be a higher priority. I can think of potential grounds for making this case: it is not very costly to provide protection, and species in Antarctica are phylogenetically more distinct or have properties that make them scientifically interesting (e.g., the ability to live in a cold and hostile environment). The authors may have more compelling lines of argument. Because Antarctica is not on the radar screens of most conservation organizations (e.g., it was largely ignored in the Aichi Targets) and does not get the attention that coral reefs, mangroves, or rainforests receive, the authors should make the case for paying attention to Antarctica in the introduction.

We thank the reviewer for this suggestion and agree that it is important to highlight why Antarctica does deserve conserving. We have added a new paragraph to the start of the introduction highlighting the primary reasons reasons (Lines 76-88), an additional sentence in the discussion highlighting the importance Antarctic ecosystem services (Lines 448-449), and a couple of sentences, as suggested, emphasising the low costs of conserving the Antarctic compared to other regions.

Given that benefits, feasibility, and costs are all uncertain, the resulting conservation return on investment that combines these elements in a ratio is likely to highly uncertain. I know it is a cheap comment by a referee and often hard for authors to actually do, but this is a case where doing sensitivity analysis given some distribution on benefits, feasibility, and cost, is really needed. Without some further analysis along these lines it is hard to know how much confidence to have in the rankings of various strategies.

We have undertaken the sensitivity analysis (as noted in response to the Reviewer's above comment). The results of the sensitivity analysis (Supplementary Figures S6 and S7) indicate that the same strategies are consistently identified as the most cost-effective regardless of the sources of uncertainty.

It is not clear to me what the conservation strategies are specifically. The descriptions in Table 1 are rather general. For example, what is involved in "Reduce and minimise impacts of existing infrastructure compared to current levels"? What does this actually entail? How much reduction is possible? What kinds of changes in ecosystems would results? What impacts would this likely have on biodiversity? How much would it cost? Even for an expert, it seems like it would be hard to answer questions about benefits, costs, and feasibility, based on such a general description of a strategy. If so, what should we really make of the return on investment numbers?

This information is provided in Supplementary Dataset S1. We apologise that this was not clearer in the first version of the manuscript and so have included an additional, explicit sentence noting this in Lines 174-175. The table details all of the actions required in each strategy to meet its objectives, as well as the costs and feasibility of each of these actions.

Reviewer #3:

[see attachment for fully formatted version]

This manuscript seeks to perform a large-scale assessment of threat management to biodiversity in Antarctica. The researchers employed a thorough expert solicitation process to do this work, with important results showcasing the most vulnerable taxa and also highlighting management paths forward. This research is timely and important; it fills a major knowledge gap while also clearly pointing to the urgent need for stronger management (and provides the useful information on the potential cost of such management actions). I thank the authors for their excellent work and look forward to seeing this manuscript in print!

We thank the reviewer for their supportive assessment of our work.

The manuscript is well written and clearly laid out with nice figures which help to illustrate the findings. Below I provide a few relatively minor suggested edits and comments.

Page 6, Line 106-109. Do we have a lack of taxonomy data or is it more accurately that we have a lack of baseline data (including abundance, distribution, etc.) regarding species?

We have expanded on this information in Line 151.

Page 6, line 120-123 (and corresponding text in the Materials and Methods section further down). It was not clear if the exercise (and metric) for intactness was something that the authored devised or if this metric had been used before by others. It would be helpful to clarify (e.g., is the authors approach for measuring taxon intactness something they drew on from other studies? Is this an established approach?)

We thank the reviewer for noting this missing detail and have added it in in the methods in Lines 771-777.

Figure 1. Nice figure. My only question/comment is: Why is there a category for “Procellariids” and a separate category for “Southern Giant Petrels”. Are the Giant petrels also considered in the Procellariid category or were they separated? (and why, it would be useful to clarify somewhere)

This is a good question – Southern Giant Petrels were considered separately. This has now been noted in Lines 657-659.

Figure 3 was not super easy to read. Any shorthand used in the figure should be explained in the caption (e.g., all stras excl IEP). On the figure, it would be useful to clearly label figure A as RCP 8.5 and B as RCP 4.5.

We have included the labels for RCP4.5 and RCP8.5 within the figure, have expanded the abbreviations themselves and have noted in the figure caption that the abbreviations are detailed in Table 1.

Throughout the text, but especially in the areas which addresses penguins and flying seabirds, there was not mention of marine-based threats such as fishing. I realize marine threats were outside the scope of the paper, but it would be useful to note this limitation (perhaps in the discussion). Particularly around the Antarctic Peninsula where krill fishing is high, there is likely direct or indirect effects on krill dependent penguins and perhaps on some flying seabirds.

We have added a couple of sentences in the methods to clarify this (Lines 669-672). Marine based threats were considered in the context of their impact on terrestrial systems (either directly on seabirds, or through seabirds interactions with the terrestrial ecosystem). Further details are available in Supplementary Table S12.

Reference to RCP 8.5 throughout the text and especially Page 27, line 506. It was a sound approach that 4.5 and 8.5 were employed (given the recommendations towards using these pathways at the time the research was conducted). However, some more recent work has also suggested the errors with RCP 8.5 (how it is economically unfeasible) and suggested that scientists do not use 8.5. See e.g., the recent work by Hausfather:
Hausfather, Z., & Peters, G. P. (2020a). Emissions–the ‘business as usual’ story is misleading. Nature 577, 618-620.
Hausfather, Z., & Peters, G. P. (2020b). RCP8. 5 is a problematic scenario for near-term emissions. Proceedings of the National Academy of Sciences, 117(45), 27791-27792.

I would suggest an edit to line 506 (perhaps considering the debate on what scenarios to use) or point to the most recent IPCC report and their rework of the pathways (to SSP). That said, having the consideration of RCP 2.6 is excellent and provides even more information and insight.

We thank the reviewer for drawing these studies to our attention. We have now added a couple of sentences to the methods discussing the nuances of RCP8.5 and have also included a sentence linking the RCPs to the SSPs (597-607).

One overall comment is that it would be useful to have the Main text clearly labeled between Introduction, Results, Discussion and Conclusion.

We have included additional headings throughout to help provide signposts to the reader.

The Conclusion is great, especially tying it to the recent declaration.

We thank the reviewer for their kind comments.
