# Supplementary material for: Threat management priorities for conserving Antarctic biodiversity
Source: PLoS Biol. 2022 Dec 22;20(12):e3001921. doi: 10.1371/journal.pbio.3001921 (PMC9778584; doi:10.1371/journal.pbio.3001921)
Supplement: Attachment — Submitted filename: Lee_PTM_Response-to-Reviewers_Rnd2.pdf [file pbio.3001921.s007.pdf]

Reviewers Comments:

Reviewer #1:

I thank the authors for the rewrite. It is now easier to understand what was being done and therefore, to comment on the manuscript. I feel that it is now at the stage where the structure and expression is beginning to make sense (but could be improved), but where it is now apparent that there are some issues in the logic that need work before the paper would be as useful as it needs to be. Nevertheless, the reason I say this is that I find the results important and worthy, and so it is all the more worthwhile to iron out the issues and communicate the results clearly before moving to publication.

We thank the reviewer for their support of our study and for their further helpful comments. We have worked through each of the comments carefully (see below) and believe that we have clarified the issues identified.

Structure and apples/oranges issues: major revision please
The paper is implicitly in two parts and one of my main recommendations is that they should be separated clearly. Climate change is the main threat to biodiversity in the Antarctic (as reinforced by the ms) but there is a limited amount that Antarctic scientists can do to halt it at <2 degrees (apart from raising the problem). Where the manuscript has value, is in showing that a number of local conservation actions, which are more under control of Antarctic staff and authorities, would still make a lot of difference. To me, those are the two obvious points. To be fair, much of the paper tacitly observes that structure, but I had to read it several times to try to tease out which parts respect it, which parts don't respect it, and what is being talked about in each section. For example, it took me three readings to deduce that cost effectiveness and complementarity probably don't include the climate action budgets, then a further reading to find out that I was wrong again after three readings. Is a strategy to influence global climate policy included, or isn't it? The answer seems to swing back and forwards in each paragraph. For example, line 325 reads "All strategies combined' and 'All strategies combined excluding policy influence' are both high cost, high benefit strategies (S3 Fig. a), whereas 'Influence external policy' is low cost...[and] high benefit (S3 Fig. a)." And yet, the table of costs right underneath line 325 contains no mention of influencing external policy. Similarly, cost-effectiveness in the following section does not seem to include influencing policy, etc.

Given that our *'Influencing external policy'* strategy does not include the global costs of climate mitigation, we did ***not*** include it in the cost-effectiveness and complementarity analyses except in one special case (ex Fig. S5 – which is discussed below, and which we have subsequently removed. Thus, it is now not included at all).

We did however include the *'Influence external policy'* strategy in the 'Feasibility and costs' discussion section as we believe it is interesting to compare the feasibility of the strategy to other strategies. We appreciate this might seem confusing and so have added an additional sentence clearly stating this (lines 377-379). We have also moved Table 2 to be underneath the 'Cost-effectiveness' section rather than above it, to help separate it from the 'Feasibility and costs' section.

I think would be an error to try to address "action by Antarctic scientists and authorities on global climate policy" and "action by Antarctic scientists and authorities on local-Antarctic conservation strategies" in the same analysis, especially when comparing cost effectiveness or complementarity.

We agree we cannot compare their cost-effectiveness or complementarity, however, we believe it was essential to consider all possible conservation actions in this study, including global climate

action. Indeed, this is one of the major findings of this study – that climate change is (not surprisingly) the greatest threat to Antarctic biodiversity, and that the Antarctic community needs to consider what they can do in terms of addressing this global issue instead of always focusing on local (less beneficial) conservation actions.

The experts interviewed would have been well placed to know what was required to address local problems, but the idea that they could have estimated precisely what they needed to do in order to achieve <2 degrees global warming stretches credulity, let alone the idea that they can put a cost on such success that allows a cost-effectiveness analysis. To compare the cost of the climate lobbying with that of the invasives program (for example) is extremely biased, since it compares a well-known practical-conservation budget with a lobbying budget that has little likelihood of being accurate and no sure prospect of knowing the true impact. (Cost, after all, is the cost of achieving the impact, and that is unknown if the impact cannot be assured).

We agree that it was not possible for this group to accurately estimate the cost of achieving <2 degrees warming and that is why we did not try. We also agree it is not fair to compare the cost of the *'Influencing external policy'* strategy with the local Antarctic conservation strategies in terms of cost-effectiveness, given that we don't know the full cost of reducing emissions. That is why *'Influencing external policy'* is not included in the cost-effectiveness analysis.

The fact that "influencing external [mostly global climate] policy" is the second-cheapest option raises particular doubt about the credibility of this part of the analysis. We already know that the budget for meeting the Paris climate commitments could run into the hundreds of billions of dollars, because many other actions beyond lobbying by a group of Antarctic stakeholders will be required. The budget for this option is therefore clearly missing stakeholders, whereas the budgets for more local actions presumably include most of the stakeholders.

The *'Influencing external policy'* strategy only includes the costs that can be undertaken by the Antarctic community, which is primarily in terms of public and policy engagement, and does not include the full global costs of mitigating climate change. This is stated in lines 177-183, lines 336-337 and again in lines 377-379. We believe this is a fair estimation of what it might cost the Antarctic community to increase their global engagement (thus 'influence global policy') and acknowledge this is only a minor cost in the great scheme of reducing global emissions. We consider it still important to talk about this cost though, as it can be a starting point for discussions amongst the Antarctic community around increasing engagement about Antarctica and climate change.

It would have been better to state the climate issue, its importance and the difficulty of achieving global political consensus, all in one section. One could then say that the experts were asked what they thought the biodiversity outcome of a particular reduction in emissions and global temperature rise would be, and even more on-a-limb, what their own policy-influencing budget might be. But it would be best to comment that by definition, the experts could not have any real sense of what the impact of their influencing would be on global climate outcomes. Then, in a separate section, move onto what the Antarctic community can more definitely achieve, what the impacts of the different conservation actions would be, their costs, cost-effectiveness and complementarity - perhaps emphasizing that none of these include the policy-influencing costs in their datasets.

We appreciate the suggestion for considering climate change separately to the other threats and local conservation strategies, however, we believe that it is important to discuss and contrast the benefits (and feasibility and costs) for all of the conservation strategies together to help compare the differences between them. As noted above, an important outcome is the emphasis that climate change *is* the greatest threat and that it should be considered when discussing conservation for

Antarctic biodiversity alongside local conservation actions. We believe this message is most clear when it is integrated and repeated throughout the manuscript, rather than presenting climate change first and dismissing it as being able to do nothing about it, and then focusing (as has been done historically) on the local conservation actions.

And to reiterate: comparing the cost effectiveness of local conservation actions with the cost effectiveness of attempts to get the global policy community to act in ways beneficial to the Antarctic (e.g. on climate change) is so far from "apples and apples" that it loses all usefulness. A global climate-change or plastics outcome is the work of thousands of paper whose expenses are not included in the cost measured here (the cost is measured to Antarctic stakeholders only. In other words, the "influence policy" cost-effectiveness/complementarity analysis divides the entire benefit by a tiny fraction of the true cost, whereas the other conservation interventions divide the full benefit by the full true cost. They are therefore not remotely comparable for the purposes of cost effectiveness or economic optimization. And yet, figure S5 shows the policy influencing and the local actions have been included in the same analysis. (Wisely, perhaps, the equivalent figure 3 in the main text does not include them).

We agree, and as noted above we did not include *'Influence external policy'* in the cost-effectiveness or complementarity analyses except in one special case. As you have identified, that was Figure S5, where it was included to contrast to Fig 3 to highlight that if you do not reduce climate change to <2 degrees than there are several species that cannot reach the complementarity threshold regardless of whether you implement all other conservation strategies. Thus, it was more to compare the number of species that can be conserved under different climate forcing scenarios rather than the costs. Given the confusion this causes though, we have now removed Fig. S5 and the associated text in the 'Complementarity' section.

As with many important papers, there is a danger of eliding over anything that may make it seem less important. I think that the message above is important and worthy and does not need any weaknesses hidden. Rather, it would benefit enormously from directly acknowledging them, and then going on to explain why the work is important anyway. Line 906 talks about " Antarctic biodiversity, which does not face the same levels of extinction and rapid human impacts as global biodiversity", and graphs in the supplementary online information show that taxa should retain ~70% intactness even under business as usual. To me, those statements should not be buried in line 900 and the online material, but be placed in the introduction, followed by a justification of why Antarctic conservation is nevertheless an issue of global importance with its own policy structure.

We thank the reviewer for noting this point and agree it should be placed more prominently in the text. We have now added an additional sentence in the 'Our approach' section stating that few Antarctic species are predicted to be at risk of extinction in this century (lines 189-191), noting that the first paragraph of the introduction reinforces why Antarctic conservation is globally important, and have added a couple of sentences in the complementarity section discussing that most species will still reach a 70% intactness threshold under the baseline scenario (lines 430-437).

The work done on confidence (that the change in intactness would lie inside the stated bounds) and on shortfalls (knowledge gaps) sounds extremely useful. It seems a shame that the manuscript has focused on some large but unsurprising outcomes such as "implementing all strategies is more expensive than implementing only some of the strategies", rather than on presenting this useful information. Some of these details contribute a lot, and yet they do not seem to feature in the main article or even appear in the results.

We agree that the shortfalls are interesting and deserve presentation. The shortfalls identified were included in the 'Uncertainty' section of the main manuscript (lines 530-535) and the tables supporting this text were contained in the Supplementary Material (S1 Table and S2 Table). To increase their visibility, we have now included a new figure in the main text (Figure 4) which summarises the number of taxa for which each shortfall was identified.

The confidence estimates help to convey uncertainty of the experts and whilst we do not discuss them directly in the main text, they are used in the sensitivity analysis (the results of which support the primary findings and are also discussed in the 'Uncertainty' section).

Impact measure: major revision please

I similarly worry about the use of "% of taxa benefiting from a conservation intervention" as the main metric. Indeed, that metric leads to some highly confusing statements. For example, *53% of species would benefit from just climate change mitigation, versus 63% of species benefiting from all possible conservation actions*, implies that climate mitigation is by far the major strategy needed for biodiversity. But this seems intuitively at odds with the following statement that *53-55% of all species will still benefit if we do not address climate mitigation and just focus on local actions*.

This confusion suggests that these percentages are potentially misinformative. I suspect that the reason both statements are possible is that any benefit, no matter how small, qualifies a taxon for inclusion in the percentage. As far as I can make out, the metric *"% of taxa affected"* doesn't distinguish between the magnitude of the impacts. So a miniscule impact on 99% of species would seem more important than a very large impact on 50% of species? If so, then such a metric does not inform conservation practice usefully. The total benefit (of a suite of conservation actions) seems a more intuitive metric to focus on, since it weights the number of taxa affected by the magnitude of the impact on each taxon. I strongly recommend the authors use this instead, even if raw percentages have the rhetorical appeal of making the impact seem bigger. Raw percentage approaches work well when seeking political election, but they are out of place in biological science. The impact should not be a simple count of the taxa what might benefit in however trivial a degree. What we need is a true impact on Antarctic biodiversity that practitioners can work with, because it is precise and properly informative of what would happen on the ground.

You are correct in that any benefit counts toward a taxon's inclusion and that the '% of taxon affected' does not distinguish between the magnitude. We agree that this does not present the whole picture, which is why we focus the second paragraph of the 'Benefits of management strategies' section on the total amount of benefit achieved for each strategy. We believe both of these combined (number of taxa that benefit + amount of benefit achieved) provide useful summaries of the benefits of each management strategy. We acknowledge this does not distinguish the magnitude of benefits for individual taxa, which is why we also include the third paragraph on individual taxa and a link to the online data which provides the results at a taxon level. There are too many taxa (and too many results) to cover the individual taxa in detail within the main text, though to help readers recognise that the magnitude of benefit can vary between taxa we have included two additional sentences in the individual taxa paragraph highlighting this (lines 305-308 and 310-312).

A more serious issue arises with the impact (benefit) being calculated as the simple arithmetic difference between two expert estimates of likely future intactness. The following section suggests that a percentage difference in intactness would be much more informative than an arithmetic one.

See response below when this is discussed in more depth.

Cost and benefits calculation (and therefore cost effectiveness and complementarity sections): major revision please

There are three issues with the cost and benefits calculation and its application to cost effectiveness and minimum set analyses: (1) using arithmetic differences to estimate benefit; (2) an apparent nonsense in measuring the cost of conservation actions that are not actually carried out, and therefore have neither cost nor benefit (3) apparent inconsistencies in whether feasibility measures are incorporated or not.

Lines 861 onward state:

861 The potential benefit per strategy was calculated as the difference between the best estimate 862 intactness value of the strategy and the intactness value of the baseline (error bars were 863 generated giving the difference between the upper/lower bound intactness values and the 864 baseline values).

So in other words, benefit is simply defined as intactness after 83 years of better conservation, minus intactness after 83 years of the same conservation as we have now? To illustrate the problem here: most people would regard a jump from 5% to 30% intactness as much more significant, in conservation terms, than improving from 70% to 95% intactness. Yet both have the same arithmetic difference. For this reason, most people would use the percentage difference, not the simple arithmetic difference.

You are correct that we used arithmetic difference and not percentage difference and you also raise a very valid point that managers *might* be more interested in, for example, a 25% increase in intactness from 5 to 30% in comparison to 70 to 95%. However, we were not comfortable making this assumption as it intrinsically weights more vulnerable taxa as more important in management analyses and we did not want to give taxa weightings. We also avoided prioritising the taxa in order of who should be managed/conserved first. We agree this is an important consideration and question, but it is not the question we were asking in this particular study. We do provide the results for all the individual taxa (expected values, benefits, and cost-effective strategies) in the supplementary and online material, so that managers interested in managing particular taxa have the information available.

Again, this section gives no information on the baseline against which a "keep it below two degrees" strategy could be compared, in terms of biodiversity outcomes.

The <2 degrees future (RCP2.6) was an outcome of the *'Influence external policy'* strategy, thus the benefit was calculated in the same way as the other strategies – the difference between the expected values of the baseline and the expected values of the strategy. This means there are RCP4.5 and RCP8.5 scenarios for the *'Influence external policy'* strategy and *'All strategies combined'* strategy the same as the other strategies (comparing to a RCP4.5 and RCP8.5 baseline). We have added an additional sentence to clarify this (lines 919-923).

To add to the confusion, S3 figure labels the benefit axis as "% benefit", rather than the arithmetic difference described everywhere else.

To avoid confusion we have relabelled the x axis to be 'Total benefit (expected intactness change, %)' and have ensured this is the same for the other relevant figures.

The cost-effectiveness of each strategy i (CE_i) was calculated as the expected benefit (sum of 876 all taxa) divided by the expected cost (C_i), where expected benefit was calculated as potential 877 benefit (B_i) multiplied by feasibility (F_i):

$CE_i = B_iF_i / C_i$

when...

Feasibility consisted of two aspects:
718 Likelihood of uptake was estimated for each action and represents the likelihood that
719 policymakers will agree to implement this action
...
Likelihood of success
722 was estimated per strategy and represents the likelihood that the strategy will meet its
723 objectives, assuming that all actions have been successfully implemented. ***Overall feasibility***
724 per strategy was calculated as a product of the two likelihoods and is provided as a probability
725 (S3 Fig.; S1 Data).) [My emphasis].

I'm afraid this seems to be a nonsense. If cost effectiveness is benefit times feasibility over cost, and feasibility is the product is the likelihood of uptake and the likelihood of success, then a strategy that is not fully taken up should not have a full cost associated with it! And yet, the cost-effectiveness measures seem to compare the full cost of the proposed strategy against the benefits of a partial implementation of it? If costings assume the action was implemented in full, then benefits should do so as well in a CBA.

Both the benefits (as calculated from the expected intactness values) and the costs were estimated by the experts assuming that the strategy was implemented in full.
In the cost-effectiveness analysis, the feasibility is then used to modify the 'pure' or potential cost-effectiveness by the feasibility. $CE_i = (B_iF_i)/C_i$ is the same as $CE = (B_i/C_i) * F_i$

We have now added a sentence (line 775) to help clarify that the estimated costings assume that all actions are successfully implemented (there is already a sentence about the benefits in lines 835-836).

This also raises the question: why, in the section immediately preceding those lines, is the benefit of an intervention apparently calculated as the simple change in intactness (**without** weighting by likelihood of success), and then recalculated **with** the weighting in the cost effectiveness section?

'Pure' benefit (without accounting for feasibility) is used in the total benefits results section because this highlights the most beneficial strategies overall, assuming that cost is not a barrier and that they are all successfully implemented and meet their objectives (i.e. in a perfect world). The cost-effectiveness analysis (and complementarity) then makes these more realistic by accounting for costs and feasibility – for this reason, both the most beneficial strategies *and* the most cost-effective strategies are highlighted as the primary results of the study (as they are different, yet both important).

Even more confusing, the next section (the complementarity analysis) then seems to add in the weighting again:

The expected intactness
913 P_{ij} for each taxon j under a given strategy i can be calculated as follows:
$P_{ij} = \bar{P}_{baseline\,j} + F_i\bar{B}$ 914 $_{ij}$,
In other words, the benefit of the intervention is weighted by the feasibility of the intervention on this occasion.

917 $\bar{B}_{ij}$ [is] the mean potential benefit across

918 contributing experts of applying strategy i for taxon j.
Again, this seems inconsistent. If this measures the benefit of applying the strategy, then it seems odd to state that you are multiplying it by the likelihood of NOT applying the strategy. This could be more precisely worded.

In summary, this all seems inconsistent and would either need to be calculated consistently, or to explain (for example) why the authors are fully costing strategies that are not going to be fully implemented.

The feasibility is incorporated into both the cost-effectiveness analysis and complementarity, and left out of the total benefits section for the reasons identified above.

In terms of 'fully costing strategies that are not going to be fully implemented' - The 'likelihood of uptake' represents the likelihood that policymakers will agree to implement the actions in a strategy, the likelihood of success represents the likelihood the strategy will meet its objectives (assuming all actions are successfully implemented). The product of the two gives the overall feasibility, as a probability, which is the overall likelihood the strategy will succeed.
The benefit estimates are based on the assumption that the strategy *is* successfully/fully implemented, which means the full costs have been incurred (strategy fully implemented: there is no option for partial implementation). Whether or not the strategy then succeeds in meeting its objectives is a different question, which is why the feasibility is used to modify the benefits.

Additionally, how fair is it to assume that the current conservation strategies (the baseline) will retain 100% of their "feasibility" over the next 83 years, whereas the alternative intervention is downgraded by its feasibility? Were the experts instructed to consider possible changes in the regularity of implementation or the possibility of current conservation becoming less/more successful between now and 2100? Or is the BAU intactness score simply a guess about biodiversity in 2100 based on current conservation activities, their success and their uptake rates? Does that guess even consider the feasibility of the current activities? Behavioural economics would suggest that practitioners engaged in current activities may have a biased appreciation of their likely future success.

This is a valid concern, though we also thought it a reasonable assumption as it seems unlikely that current conservation actions (e.g., biosecurity procedures, non-native species management) will be much reduced given they are not consistently or comprehensively implemented anywhere across the continent currently. Antarctic Treaty Parties are also required to maintain some minimum standards under the Antarctic Treaty (though the standard varies based on the Party).

The experts were not instructed to consider possible changes in the regularity of implementation of current conservation actions as we considered this would further complicate a process that was already sufficiently complex (also given that current conservation actions are irregularly implemented, considering a change to the regularity of current actions seems a minor concern). By definition we considered that current conservation actions would not become more or less successful by 2100 as they are based on the current success rates – we consider that for them to increase in success, then additional action would be needed to be implemented and if there are additional actions applied then this falls under the realm of one of the management strategies.

The baseline scores are an estimate of the intactness of taxa in 2100 given the threats they will face over that time period but assuming that the level of conservation actions are the same as they are in the current. We believe the experts have a good understanding of their success and uptake rates. We don't consider feasibility to be a major factor for current conservation actions (as mentioned

above) as they are already implemented/used and so we know they are feasible with some certainty.

Complementarity section: major question

Conveniently the P_{ij} can be assessed
919 under different persistence thresholds and provide a binary matrix T such that an element T_{ij}
920 takes value 1 if the expected persistence of taxon j under strategy i (P_{ij}) is above the designated threshold and 0 otherwise.
The complementarity problem is usually formulated as maximising the number of taxa above the designated threshold for a given budget (Carwardine et al. 2019). Here, we chose to minimise the cost of securing a given number of taxa above threshold.
The first set of decision variables y_i determines if a strategy $i \in S$ is selected (y_i = 1) or not (y_i = 0). If strategy i is selected, the taxon j with values of T_{ij}=1 is assumed secured. The second set of auxiliary decision variables x_{ij} identifies the taxon j secured by strategy i – these decision variables ensure we reach the target of a given number of taxa to secure (identified by Target).
(c2): For the purpose of the optimisation, only one strategy can count towards securing a taxon above the threshold.

This is a somewhat odd specification of the problem, again. To reiterate: if the conservation benefit is multiplied by feasibility, and feasibility includes the likelihood of not implementing the strategy, then it would again seem that the optimized investment profile currently includes paying for things which were not actually done!

See above for answer to this topic.

Perhaps more important is the way the specification seems to implement the measures one at a time and then assume that species intactness only changes as a result of the implemented strategy. (Thus, in the first step in Figure S5, it seems that only "controlling human activities" would be implemented). But does this take account of the negative impact of inaction on the other fronts. For example, if "control human activities" is implemented, the program calculates that a large number of taxa will achieve a certain intactness threshold based purely (in my reading) on the benefit of controlling human activities. But if the same taxa are likely to lose intactness from e.g. invasives, then intactness would correctly be calculated as the net outcome, including both the benefit of the implemented action and the losses occurring due to any non-implemented action. Thus, I suspect the correct specification would be not 'expected persistence under strategy i', but rather 'expected persistence under the full suite of strategies, including those not implemented', i.e. sum of all Pij where some strategies are set to a marginal zero. If my description is not the correct one, I would like to see some text clarifying how the net impact of all actions and inactions is being calculated. Is it that the "cost of inaction" is implicitly contained in the baseline (as I suspect is happening)? If so, then please clarify that inaction on some fronts negatively affects intactness at the same time that action on other fronts positively affects it.

Your suspicion is correct, the 'cost of inaction' is implicitly contained in the baseline, as the baseline is the future intactness of the taxa subject to *all* threats. We apologise if this was not obvious in your first reading of the complementarity methods. We have added an explicit sentence highlighting this in lines 980-985.

Additionally, there is a clash between what many readers will assume is "complementarity" in conservation actions, and what the integer linear programming procedure means by the word. For

many readers, "complementary" actions might reinforce each other, making them more effective, but the ILP process described does not seem to incorporate such "sum of the whole is greater than the parts" effects, despite the text referring to them (e.g. action achieving climate improvement would reduce the need for action on invasives). There will also be some redundancy, for example if protected areas contribute to vegetation conservation. Quite a lot more needs to be done by the authors here, either to account for how multiple conservation actions work together in the modelling, or to discuss how a failure to account for that could influence the conclusions.

You are correct in that we did not consider interactions between the conservation strategies, except for the two combined strategies (*'All strategies combined'* and *'All strategies combined excluding influence external policy'*) where the experts did consider that all the strategies would all be implemented together and estimated the maximum total benefit possible.

However, this is not an issue for either the complementarity or cost-effectiveness analysis because the benefits of the strategies are always considered independently (and the combined strategies already have this issue accounted for). We do not sum benefits across strategies for the complementarity analysis (or CE analysis) as you have suggested. The complementarity analysis identifies the most taxa that can be conserved under different budgets. It is threshold based and identifies the number of taxa that will reach threshold *x* when strategy *y* is implemented. If multiple strategies help the same taxa to reach the threshold, then the strategy that is cheaper is selected. If multiple strategies help different taxa to reach the threshold, then they are complementary (in the general definition of 'complementary', that these strategies implemented together will complement each other because they don't do the same thing).

For example, in Figure 3 under RCP8.5, to reach a 90% threshold with a budget of $250 M – the optimal strategies to invest in are *'Manage new infrastructure'* and *'Protecting areas'* and this increases the number of taxa that can reach the threshold to 27 (from a baseline of 24). *'Manage new infrastructure'* would increase the number of taxa by two (podoromorpha springtails and southern giant petrels) and *'Protecting areas'* would also increase the number of taxa by two (entomobryomorpha springtails and southern giant petrels). Southern giant petrels can reach the threshold independently with either strategy (the benefits are not summed together), and whilst their may be some redundancy for the southern giant petrel by implementing both strategies, there is not redundancy overall because both strategies are still required – one helps podoromorpha springtails and one entomobryomorpha springtails. If you had a larger budget, then you could instead implement *'Manage non-natives species'* and this would help the same three taxa to reach the threshold plus an additional three. But if you only have $250 M then this is the best you can achieve.

We have added two paragraphs in the complementarity methods section (lines 1018-1041) to clarify that interactions between strategies are not considered and to describe how this may differ from reality. We also added a sentence in the figure legend of Figure 3 to help with interpretation of the complementarity analysis (lines 444-447).

Discounting of future costs (but not discounting of future benefits?): major question/revision:

As far as I can make out, the annual costs are calculated over exactly 83 years and then suddenly are set to zero in 2100. Moreover, it seems as if discounting has been applied to future costs but not to future benefits. (Benefits are essentially the delta intactness assumed achieved by a strategy in 2100). Typically, economists discount the future flow of benefits, rather than costs but not benefits. It is therefore odd to discount something not normally discounted, not discount something that is normally discounted, and then compare the two anyway.

The first question here, however, is whether applying a discount rate to future costs is appropriate, unbiased or even useful at all. On appropriateness, discounting future costs assumes that an investor in conservation is looking for a financial return, which is unusual for an Antarctic conservation situation. Government costing exercises typically want to know the simple cost, not a discounted cost, because future discounting of costs would be such an odd thing to do. It's therefore not clear how appropriate the future discounting of cost is, on principle.

As regards to bias, it would appear that the authors describe a "social discount rate". Again, social discount rates are usually applied to benefits, including non-financial benefits such as future outcomes for biodiversity. But the cost-benefit analysis is clearly biased if one discounts the costs but not the benefits.

Discounting costs are important when the payment schedules of actions differ over time to allow fair comparison of action costs – we do not have a basis for benefits accruing differently over time for the benefits metric. It would be unnecessarily complex and result in a less easily interpretable metric. It is standard in almost all conservation prioritisation approaches to not discount benefits. To ensure the reader is aware we did not discount future benefits, we have added a sentence in lines 927-929.

Discounting costs also does not assume that an investor in conservation is looking for a financial profit, instead assuming that an investor is keen to make the most of their limited conservation dollars. When planning for long term conservation goals, an investor might invest in a trust (or equivalent) ensuring that money set aside now will at least increase with inflation and be able to cover the future conservation costs. This is why we use ‘present value’, as the costs are equivalent to those of today.

As regards to usefulness: if the main analysis is about the relative cost, cost effectiveness and cost complementarity of the various strategies, then the outcome will be the same irrespective of how these are discounted, unless there are major differences in how costs unfold over future years. The future trajectory of costs is neither described by the authors, nor justified as a reason for the discounting. This would need to be remedied before a reviewer could even make a judgement on whether discounting of costs was genuinely useful.

As noted above, discounting is important when payment schedules differ over time, and that is the case over the 83-year time frame considered here. Some strategies require a substantial investment within the first 0-5 years (e.g. when undertaking baseline biodiversity surveys), whilst others have recurring costs over time (e.g. updating ASPA management plans every five years), and still others are expected to have increased costs over time as climate warms (e.g. increased likelihood of non-native species establishment over time means experts estimated more non-native species management would be needed later in the timeframe). The costs for each action and the timeframes for when they need to be implemented are detailed in Dataset S1.

We have added several sentences in the methods to justify the importance of discounting costs and to provide examples of strategies with different cost schedules (lines 779-785).

If cost discounting seems relatively uninformative, then this raises a concern that the actual cost is vastly understated because by 2100, costs presumably fall to almost zero (after 83 years of compound discounting at 2%*)*, which would be a most questionable conclusion. This indeed becomes apparent when the authors mention, in a brief parenthesis, that without discounting, the cost would jump from $1.92 billion to $3.69 billion i.e. it would pretty much double.

Costs do not fall to almost zero at the 83-year time point (it depends where the actions are spread out on the timeline), and we do not set them to zero or assume that they will be zero after 2100. We did need to set a stopping point and a timeframe for the actions to be applied over (as is standard in conservation problems), as we could not estimate costs or benefits going forward indefinitely.

I do rather suspect that it is the question of how to spread out capital costs that influences some of the authors' thinking around discounting. But another way to spread capital cost is to assume amortization on, effectively, a loan to cover initial set-up costs for a strategy.

You are correct, considering how the capital costs are borne is one factor. A loan is one option, though not an ideal one for conservation projects. We instead chose to use discounting, recognising that less money would be required overall, if at the start of the project enough money is made available for the whole project – incorporating the 'discount' that is applied by initially setting aside the money for future actions in a trust etc.

Overall, I would say that the authors either need to discount future benefits and future costs in a consistent way, or they could quite feasibly drop the discounting of future costs completely, since it is atypical and of unclear usefulness in the comparisons (e.g. the ratio of two discounted values is similar to the ratio of two undiscounted values, and most of the paper is about such ratios).

Whilst the ratio of discounted and undiscounted values do not vary hugely (which as you noted would then not have a large impact on the CE results etc), we still consider discounting to be an important consideration whilst planning conservation actions over a long timeframe and one that is commonly applied in conservation science.

Future values in general and setting costs to zero after exactly 83 years.
On a broader point: how can we possibly estimate the costs of actions up to 83 years in the future? Did the authors and experts largely assume current costs, ignoring how these would change in the future? For example, if climate change causes more invasive species in the second half of the century, that would increase the future costs of combating invasives. And if human population and wealth increase (as is likely), then both tourism and other pressures would increase over time. Any such future change would alter the outcomes of all the cost and cost effectiveness analyses performed here, but that possibility does not seem to be addressed in the sensitivity testing.

Whilst we did assume current costs in terms of the $ value (e.g. 1FTE now costs the same as 1FTE in the future), the experts did consider how the amount of actions or effort would change over time. A prime example is as you suggested, and as noted above, that more effort is required to combat non-native species in the second half of the century (requiring an increased quantity of management actions). Increased costs are also estimated for managing and protecting species (more species impacted and at greater magnitudes later in the century, thus requiring increased individual management), and protecting areas (because as more ASPAs are designated, then there is more monitoring and revising of management plans to be done).

Minor points

However, for
303 those species that are predicted to benefit with climate change, at least initially (e.g. gentoo
304 penguins), they benefit less with the milder RCP2.6 scenario in contrast to RCP4.5 or RCP8.5.
17
Thus, it appears that they have reduced intactness 305 with the RCP2.6 'Influence external policy'

I realise that counterintuitive results need to be explained but why not present the main patterns, then at the end, say that some results may seem surprising e.g. Gentoo penguins doing better and better and climate change gets worse and worse, and explain what lies behind those surprises. The way the text structure is set up currently, it feels like the anomalous is being given the same weight as all the main results.

We believed it important to note anything that seemed surprising and considering there are quite a few taxa predicted to benefit from climate change, we did not consider this really an anomalous result. By placing it at the end of the benefits section we do consider it to be at the end and after the main results (whilst still remaining in a relevant place in the text).

337 Considering multiple social discount rates, where future costs are discounted to present-day
338 values (PVs; see Materials and Methods), combining all strategies was consistently estimated
339 to be the most expensive strategy
Unless different discounting rates were applied to different strategies, then the comparative cost of the strategies has no connection to the discount rates. The start of this sentence therefore seems to be a red herring.

We agree and have deleted the first part of the sentence.

Line 376: the first paragraph clearly belongs to the previous section on cost, not to the new section on cost effectiveness. Overall, there still seems to be some work to be done on clearly structuring the manuscript. The conclusions are important, so it is really worth getting this right!

We have moved this paragraph to the previous section.

Section on Implementing the Conservation Actions:
This is well written, eminently sensible and should provide the core of what this paper is about. Perhaps a mention of the problems of financing a solution when one is not dealing with a single nation's sovereign territory would be a useful addition

We thank the reviewer for this helpful suggestion and have added a sentence in lines 357-359.

the strategies were reasonably robust
519 to all sources of uncertainty.
Not so, and this is unscientific. They are robust to precisely the uncertainties that the authors tested, no more. It would be more useful and honest to state at this point in the ms what uncertainties were tested for, precisely.

We have reworded as “to all sources of uncertainty considered here” (line 548). The uncertainties that are considered (cost, feasibility, intactness) are listed earlier in the sentence.

522 despite the uncertainties in our experts estimates,
an apostrophe is needed here

Thank you, this has been added.

the top three most
523 beneficial, or most cost-effective strategies are the same regardless of RCP, region or whether
524 upper or lower bound is used (see Fig. 1; Table 2; S5, S6 Figs.; S4, S5 Tables).

Why not restate them briefly? Indeed, by this point, I had no memory of what the "top three most beneficial strategies" might have been - except that reducing climate change would be most important.

We have done this (lines 555-557).

611 2100 (UNFCCC 2015). Therefore, RCP2.6, the low emissions scenario and the only one to
612 keep global warming to <2 °C (IPCC 2013; Gasser et al. 2015), was used to assess the
613 'Influence external policy' and 'All Strategies Combined' strategies

Does this mean that the experts were told to assume that the action 'influencing external (global) climate policy' would lead to the earth limiting climate change to a maximum two-degree temperature increase globally, and to estimate how much more intact the biota would be if we achieved that? If so, what is the baseline against which the intactness was measured? How are the benefits calculated?

See comment above where this was discussed (and lines 919-923). You are correct in your interpretation that the experts estimated how much more intact the biota would be under RCP2.6. This intactness was compared against both the RCP4.5 and RCP8.5 baselines.

Later, the text says *"if.. the world were committed to >2 °C warming".* This is highly imprecise and does not give a quantitative baseline against which to compare. How much warming above two degrees? It makes a lot of difference.

RCP2.6 is the only scenario we used that keeps warming to <2 degrees, so either of the other two strategies (RCP4.5 or RCP8.5) are above 2 degrees. This has been clarified in line 745.

764 it was assumed that costs and feasibility would remain the same for
765 both RCP4.5 and RCP8.5.

This seems like a poor assumption, requiring more justification and more exploration of the consequences of this decision.

This was a necessary decision as it was not possible to ask the experts to provide two sets of costings and feasibility on top of everything else they had to estimate. This has been noted in line 812.

S4 Figure and elsewhere: it's confusing to label the cost as "present value". Please label it as "cost" and if necessary, use brackets after that to indicate that the cost has been discounted. Also, these figures seem to have returned to using the total cost (a total in which annual spending is arbitrarily halted after exactly 83 years) that was the subject of an objection in the previous review.

If the authors indeed wish to apply a discount rate to future annual costs (something that is more typically applied to benefits), then at minimum, they need to specify in all figures whether they are using the dollar values for a particular year (constant dollars 20XX). Otherwise, inflation makes a mockery of the discounting.

Each of these elements were discussed above. Present value is used as the costs are equivalent to present-day costs (in 2017). Annual spending is not arbitrarily halted after 83-years, a timeframe was necessary for the project.

We have noted in the figure legend of Figure 3 that present value refers to dollar values in 2017 (lines 448-449).

S5 Figure: here it suddenly becomes apparent that a cost, and a cost effectiveness, has been placed upon "influence external policy" - something that after three readings, I had deduced had not been done because it would not remotely be a fair or unbiased comparison. Examination of the data table shows that this cost largely involves employing a phalanx of communications or back-office professionals to influence COP discussions and public opinion on climate and plastics. When this is put into a complementarity graphic, it really brings home how heroic the assumptions have to be to make this work: expected cost effectiveness is investment divided by likely degree of success, but what is the likelihood of success for influencing of global COP outcomes? In other words, is it even remotely reasonable to estimate the impact of communicators on a global climate goal (ignoring all the other parties trying to achieve similar things), and then confidently calculate their likely cost effectiveness? My own feeling is that no, it is not reasonable to make such giant leaps of faith in assuming what the communicators could achieve for global climate outcomes.

As noted above this figure was kept primarily to compare how many more taxa could be secured if climate change could be addressed. We have now removed this figure from the supplementary.

We have also added an additional sentence noting the potential for Antarctic Treaty Parties to contribute to global meetings (lines 467-470).

In the graphic itself, the discontinuous steps need more explanation. For example, "new infra + protecting areas" seems to cover a range of possible budgets from about $200 million to about $800 million, which is likely to be highly confusing to the reader.

There is only a single budget point for each strategy/sets of strategies, so if you have a budget above the amount *x* required, you still only invest *x* as this is the best use of your money. If you have enough budget to get to the next step, then you invest in that strategy/strategies instead.

An example has been provided in the figure legend to help readers with interpretation (lines 444-447).

There is a surprising different between RCP4.5 and RCP8.5, in that protecting areas is an efficient strategy in one but not the other. This also requires explanation.

Protecting areas is identified as an optimal strategy in both RCP4.5 and RCP8.5, though in RCP8.5 it should be implemented together with managing new infrastructure. Differences between the two RCPs are due to differences in the amount of benefit predicted under the two.

lower or upper bounds for the cost or feasibility estimates were not
973 elicited.

This seems quite an unusual omission, especially for budget estimation. Moreover, it seems slightly inconsistent with the online material, where it is stated:

" Cost Range
A number of actions have a cost range depending on various factors outlined in the action details/ assumptions made (eg. the potential number of invasive species that could establish, or whether it will take 2 or 10 FTE for a job). When a range is presented in the cost column, it is the range of minimum-maximum price expected for that action"

Whilst the experts did provide a range for some actions (where there was more uncertainty), the average was taken for use in the analysis. The analyses were already complex without adding

additional parameters. Variation in costs and feasibility and how these might impact the analyses were considered in the sensitivity analysis (and found to have little impact).

S1Data:
The table should be given a table identifier (e.g. Table S1).

‘Data’ is the Plos Biology identifier for a dataset.

If there are two likelihoods in the feasibility score, why is only one (likelihood of uptake) included in the table, and the arguably more interesting one (likelihood of success in having the expected impact) is omitted?

Likelihood of success was estimated at a strategy level (whilst likelihood of uptake was estimated at an action level), thus the estimates were provided in the first column of the table (underneath the strategy name in brackets). We have now converted this table to an excel spreadsheet for ease of use and have now added in an additional column for likelihood of success so that it is easier to find.

Reviewer #2:

I think the authors have done a good job of responding to my comments. I continue to think that the paper is well written and well executed and is probably the strongest paper that could be written on this subject. Given that the editorial decision is that this topic is of high interest, I think the paper should be published.

We thank the reviewer for their support and appreciate their acknowledgement of the changes made in the first round of review. We believe the manuscript is much improved for those useful comments.

The introduction does a much better job of motivating the importance of conserving Antarctic biodiversity. The treatment of climate change has been clarified and improved. I agree with the authors that climate change is the primary threat to Antarctic biodiversity and deserves to be fully discussed. I appreciate, however, that the authors do not include climate change in the discussion of cost-effective strategies, where costs refer more directly to actions undertaken by the Antarctic community.

Thank you.

My main concern on the previous was about the weakness of the underlying information base, which is an unavoidable issue. The authors have undertaken a sensitivity analysis to show that the cost-effectiveness rankings "were reasonably robust to all sources of uncertainty." I couldn't find figures S5 and S6 in my document, so I was not able to verify whether I agree that the rankings are reasonably robust or not. Providing a sentence or two saying what changes in the ranking under what circumstances would help. I think their main point that the top choices remain top choices for a wide range out of outcomes is the right result to emphasize. The description of the sensitivity analysis starting on line 961 was somewhat opaque (at least to me). I did not understand what the authors meant when they said "The intactness estimates given by the experts were standardised using linear extrapolation of the lower, best, and upper bounds, and confidence interval to fit 80% credible bounds around individuals' best estimates." I think the authors have done a credible sensitivity analysis, but I would ask that they provide a bit clearer explanation of what they did.

We apologise that the revised supplementary material was not provided to you with the updated manuscript.
We have provided an additional sentence to note some of the changes that could occur in the rankings (lines 550-552).
We have also clarified the sensitivity methods and added additional details to aid readers in interpretation of the sensitivity methods: lines 1045-1065 (particularly lines 1046-1050).

One last small point that perhaps could be worked into the paper is about experts and uncertainty. It has been found in the literature that experts often are over-confident and place too small uncertainty bounds around their estimates. This would inject a further note of caution into interpreting the results.

We have added a sentence into the 'Uncertainty' section to note this (lines 538-540).
