# Supplementary material for: Threat management priorities for conserving Antarctic biodiversity
Source: PLoS Biol. 2022 Dec 22;20(12):e3001921. doi: 10.1371/journal.pbio.3001921 (PMC9778584; doi:10.1371/journal.pbio.3001921)
Supplement: Attachment — Submitted filename: Lee_PTM_Response-to-Reviewers_Rnd3.pdf [file pbio.3001921.s008.pdf]

Lee et al. - Priority Threat Management – Response to Reviewers #3

Academic Editor's Comments:

a) The Academic Editor says "I appreciate the intention to not assume whether managers might be interested more in percentage rather than arithmetic differences in intactness (pg 5 of the response). However, the authors are still - albeit implicitly - making an assumption about what managers might be interested in. One sentence acknowledging the difference between interpretations based on arithmetic vs percentage differences could help round off this issue."

We agree, we are still making an assumption, and thank the editor for noting this. We have added a few sentences into the methods section to acknowledge that we are using the arithmetic difference and how using a percentage change method might vary the results (and when it might be useful to consider). See Lines 920-929.

b) The Academic Editor has a second request: "The authors acknowledge the concern about the regularity of implementation by 2100 might change (pg 7 of review), but it is unclear how this issue has been addressed in the text. Again, can there be one sentence identifying this issue and briefly explaining why it might/might not influence the interpretation of the results? I wonder if this goes into the "Uncertainty" section and maybe it's already sort of touched on at line 540."

We have added a sentence acknowledging that we did not consider the change in regularity of actions relating to the baseline *'Business as usual'* strategy (see Lines 733-737). Current conservation actions (which continue under the baseline) are currently implemented irregularly and a change in the frequency of implementation constitutes one of the alternative management strategies considered. The other management strategies did consider regularity of implementation - this is identified in Lines 786-791 whilst discussing discounting.

Editor's Comments:

c) Please hyphenate "threat-management" in the Title (as you have in the Abstract).

This was a typo in the abstract (thank you for picking it up), as we did not intend 'threat management' to be hyphenated, as it has not been in previous priority threat management works. I have removed the hyphen from the abstract, though understand if the journal style is to hyphenate throughout.

d) Please address my Data Policy requests below; specifically, we need you to supply the numerical values underlying Figs 1AB, 2AB ,3AB, 4, S1AB, S2, S3ABC, S4ABC, S5ABC, S6, either as a supplementary data file or as a permanent DOI'd deposition. Note that your current provision under the Australian Antarctic Data Centre 1. Is not currently accessible for me to check, and 2. Sounds like it may not contain the numerical values displayed in the Figures.

We have now included all of the numerical values underlying the figures in an excel workbook with one tab per figure (S2 Data).

You are correct, the data on the Australian Antarctic Data Centre (AADC) is the averaged expert values, so does not contain the numerical values displayed in the figures (though they are used to derive these values). The AADC has embargoed the data until publication, though if you would like to check these data, please let me know and I can email through a copy.

e) Please cite the location of the data clearly in all relevant main and supplementary Figure legends, e.g. “The data underlying this Figure can be found in S1 Data” or “The data underlying this Figure can be found in https://doi.org/XXXX”

This has been done.

As you address these items, please take this last chance to review your reference list to ensure that it is complete and correct. If you have cited papers that have been retracted, please include the rationale for doing so in the manuscript text, or remove these references and replace them with relevant current references. Any changes to the reference list should be mentioned in the cover letter that accompanies your revised manuscript.

I have carefully checked the reference list to ensure all of the references referred to in-text are listed (and vice versa). To the best of my knowledge, none of the cited papers have been retracted.
