# Supplementary material for: Threat management priorities for conserving Antarctic biodiversity
Source: PLoS Biol. 2022 Dec 22;20(12):e3001921. doi: 10.1371/journal.pbio.3001921 (PMC9778584; doi:10.1371/journal.pbio.3001921)
Supplement: Attachment — Submitted filename: PBIOLOGY_Review.docx [file pbio.3001921.s004.docx]

PBIOLOGY-D-21-02370R1

This manuscript seeks to perform a large-scale assessment of threat management to biodiversity in Antarctica. The researchers employed a thorough expert solicitation process to do this work, with important results showcasing the most vulnerable taxa and also highlighting management paths forward. This research is timely and important; it fills a major knowledge gap while also clearly pointing to the urgent need for stronger management (and provides the useful information on the potential cost of such management actions). I thank the authors for their excellent work and look forward to seeing this manuscript in print!

The manuscript is well written and clearly laid out with nice figures which help to illustrate the findings. Below I provide a few relatively minor suggested edits and comments.

Page 6, Line 106-109. Do we have a lack of taxonomy data or is it more accurately that we have a lack of baseline data (including abundance, distribution, etc.) regarding species?

Page 6, line 120-123 (and corresponding text in the Materials and Methods section further down). It was not clear if the exercise (and metric) for intactness was something that the authored devised or if this metric had been used before by others. It would be helpful to clarify (e.g., is the authors approach for measuring taxon intactness something they drew on from other studies? Is this an established approach?)

Figure 1. Nice figure. My only question/comment is: Why is there a category for “Procellariids” and a separate category for “Southern Giant Petrels”. Are the Giant petrels also considered in the Procellariid category or were they separated? (and why, it would be useful to clarify somewhere)

Figure 3 was not super easy to read. Any shorthand used in the figure should be explained in the caption (e.g., all stras excl IEP). On the figure, it would be useful to clearly label figure A as RCP 8.5 and B as RCP 4.5.

Throughout the text, but especially in the areas which addresses penguins and flying seabirds, there was not mention of marine-based threats such as fishing. I realize marine threats were outside the scope of the paper, but it would be useful to note this limitation (perhaps in the discussion). Particularly around the Antarctic Peninsula where krill fishing is high, there is likely direct or indirect effects on krill dependent penguins and perhaps on some flying seabirds.

Reference to RCP 8.5 throughout the text and especially Page 27, line 506. It was a sound approach that 4.5 and 8.5 were employed (given the recommendations towards using these pathways at the time the research was conducted). However, some more recent work has also suggested the errors with RCP 8.5 (how it is economically unfeasible) and suggested that scientists do not use 8.5. See e.g., the recent work by Hausfather:

Hausfather, Z., & Peters, G. P. (2020a). Emissions–the ‘business as usual’ story is misleading. *Nature* 577, 618-620.

Hausfather, Z., & Peters, G. P. (2020b). RCP8. 5 is a problematic scenario for near-term emissions. *Proceedings of the National Academy of Sciences*, *117*(45), 27791-27792.

I would suggest an edit to line 506 (perhaps considering the debate on what scenarios to use) or point to the most recent IPCC report and their rework of the pathways (to SSP). That said, having the consideration of RCP 2.6 is excellent and provides even more information and insight.

One overall comment is that it would be useful to have the Main text clearly labeled between Introduction, Results, Discussion and Conclusion.

The Conclusion is great, especially tying it to the recent declaration.
