# Supplementary material for: Threat management priorities for conserving Antarctic biodiversity
Source: PLoS Biol. 2022 Dec 22;20(12):e3001921. doi: 10.1371/journal.pbio.3001921 (PMC9778584; doi:10.1371/journal.pbio.3001921)
Supplement: Attachment — Submitted filename: Rev2 round2 comments.docx [file pbio.3001921.s006.docx]

**Review: original text in italics, reviewer comments in plain text font.**

I thank the authors for the rewrite. It is now easier to understand what was being done and therefore, to comment on the manuscript. I feel that it is now at the stage where the structure and expression is beginning to make sense (but could be improved), but where it is now apparent that there are some issues in the logic that need work before the paper would be as useful as it needs to be. Nevertheless, the reason I say this is that I find the results important and worthy, and so it is all the more worthwhile to iron out the issues and communicate the results clearly before moving to publication.

**Structure and apples/oranges issues: major revision please**

The paper is implicitly in two parts and one of my main recommendations is that they should be separated clearly. Climate change is the main threat to biodiversity in the Antarctic (as reinforced by the ms) but there is a limited amount that Antarctic scientists can do to halt it at <2 degrees (apart from raising the problem). Where the manuscript has value, is in showing that a number of local conservation actions, which are more under control of Antarctic staff and authorities, would still make a lot of difference. To me, those are the two obvious points. To be fair, much of the paper tacitly observes that structure, but I had to read it several times to try to tease out which parts respect it, which parts don't respect it, and what is being talked about in each section. For example, it took me three readings to deduce that cost effectiveness and complementarity probably don't include the climate action budgets, then a further reading to find out that I was wrong again after three readings. Is a strategy to influence global climate policy included, or isn't it? The answer seems to swing back and forwards in each paragraph. For example, line 325 reads "All strategies combined’ and ‘All strategies combined excluding policy influence’ are both high cost, high benefit strategies (S3 Fig. a), whereas ‘Influence external policy’ is low cost...[and] high benefit (S3 Fig. a)." And yet, the table of costs right underneath line 325 contains no mention of influencing external policy. Similarly, cost-effectiveness in the following section does not seem to include influencing policy, etc.

I think would be an error to try to address "action by Antarctic scientists and authorities on global climate policy" and "action by Antarctic scientists and authorities on local-Antarctic conservation strategies" in the same analysis, especially when comparing cost effectiveness or complementarity. The experts interviewed would have been well placed to know what was required to address local problems, but the idea that they could have estimated precisely what they needed to do in order to achieve <2 degrees global warming stretches credulity, let alone the idea that they can put a cost on such success that allows a cost-effectiveness analysis. To compare the cost of the climate lobbying with that of the invasives program (for example) is extremely biased, since it compares a well-known practical-conservation budget with a lobbying budget that has little likelihood of being accurate and no sure prospect of knowing the true impact. (Cost, after all, is the cost of achieving the impact, and that is unknown if the impact cannot be assured).

The fact that "influencing external [mostly global climate] policy" is the second-cheapest option raises particular doubt about the credibility of this part of the analysis. We already know that the budget for meeting the Paris climate commitments could run into the hundreds of billions of dollars, because many other actions beyond lobbying by a group of Antarctic stakeholders will be required. The budget for this option is therefore clearly missing stakeholders, whereas the budgets for more local actions presumably include most of the stakeholders.

It would have been better to state the climate issue, its importance and the difficulty of achieving global political consensus, all in one section. One could then say that the experts were asked what they thought the biodiversity outcome of a particular reduction in emissions and global temperature rise would be, and even more on-a-limb, what their own policy-influencing budget might be. But it would be best to comment that by definition, the experts could not have any real sense of what the impact of their influencing would be on global climate outcomes. Then, in a separate section, move onto what the Antarctic community can more definitely achieve, what the impacts of the different conservation actions would be, their costs, cost-effectiveness and complementarity - perhaps emphasizing that none of these include the policy-influencing costs in their datasets.

And to reiterate: comparing the cost effectiveness of local conservation actions with the cost effectiveness of attempts to get the global policy community to act in ways beneficial to the Antarctic (e.g. on climate change) is so far from "apples and apples" that it loses all usefulness. A global climate-change or plastics outcome is the work of thousands of paper whose expenses are not included in the cost measured here (the cost is measured to Antarctic stakeholders only. In other words, the "influence policy" cost-effectiveness/complementarity analysis divides the entire benefit by a tiny fraction of the true cost, whereas the other conservation interventions divide the full benefit by the full true cost. They are therefore not remotely comparable for the purposes of cost effectiveness or economic optimization. And yet, figure S5 shows the policy influencing and the local actions have been included in the same analysis. (Wisely, perhaps, the equivalent figure 3 in the main text does not include them).

As with many important papers, there is a danger of eliding over anything that may make it seem less important. I think that the message above is important and worthy and does not need any weaknesses hidden. Rather, it would benefit enormously from directly acknowledging them, and then going on to explain why the work is important anyway. Line 906 talks about " Antarctic biodiversity, which does not face the same levels of extinction and rapid human impacts as global biodiversity", and graphs in the supplementary online information show that taxa should retain ~70% intactness even under business as usual. To me, those statements should not be buried in line 900 and the online material, but be placed in the introduction, followed by a justification of why Antarctic conservation is nevertheless an issue of global importance with its own policy structure.

The work done on confidence (that the change in intactness would lie inside the stated bounds) and on shortfalls (knowledge gaps) sounds extremely useful. It seems a shame that the manuscript has focused on some large but unsurprising outcomes such as "implementing all strategies is more expensive than implementing only some of the strategies", rather than on presenting this useful information. Some of these details contribute a lot, and yet they do not seem to feature in the main article or even appear in the results.

**Impact measure: major revision please**

I similarly worry about the use of "% of taxa benefiting from a conservation intervention" as the main metric. Indeed, that metric leads to some highly confusing statements. For example, *53% of species would benefit from just climate change mitigation, versus 63% of species benefiting from all possible conservation actions*, implies that climate mitigation is by far the major strategy needed for biodiversity. But this seems intuitively at odds with the following statement that *53-55% of all species will still benefit if we do not address climate mitigation and just focus on local actions*.

This confusion suggests that these percentages are potentially misinformative. I suspect that the reason both statements are possible is that any benefit, no matter how small, qualifies a taxon for inclusion in the percentage. As far as I can make out, the metric *"% of taxa affected"* doesn't distinguish between the magnitude of the impacts. So a miniscule impact on 99% of species would seem more important than a very large impact on 50% of species? If so, then such a metric does not inform conservation practice usefully. The total benefit (of a suite of conservation actions) seems a more intuitive metric to focus on, since it weights the number of taxa affected by the magnitude of the impact on each taxon. I strongly recommend the authors use this instead, even if raw percentages have the rhetorical appeal of making the impact seem bigger. Raw percentage approaches work well when seeking political election, but they are out of place in biological science. The impact should not be a simple count of the taxa what might benefit in however trivial a degree. What we need is a true impact on Antarctic biodiversity that practitioners can work with, because it is precise and properly informative of what would happen on the ground.

A more serious issue arises with the impact (benefit) being calculated as the simple arithmetic difference between two expert estimates of likely future intactness. The following section suggests that a percentage difference in intactness would be much more informative than an arithmetic one.

**Cost and benefits calculation (and therefore cost effectiveness and complementarity sections): major revision please**

There are three issues with the cost and benefits calculation and its application to cost effectiveness and minimum set analyses: (1) using arithmetic differences to estimate benefit; (2) an apparent nonsense in measuring the cost of conservation actions that are not actually carried out, and therefore have neither cost nor benefit (3) apparent inconsistencies in whether feasibility measures are incorporated or not.

Lines 861 onward state:

*861 The potential benefit per strategy was calculated as the difference between the best estimate*

*862 intactness value of the strategy and the intactness value of the baseline (error bars were*

*863 generated giving the difference between the upper/lower bound intactness values and the*

*864 baseline values).*

So in other words, benefit is simply defined as intactness after 83 years of better conservation, minus intactness after 83 years of the same conservation as we have now? To illustrate the problem here: most people would regard a jump from 5% to 30% intactness as much more significant, in conservation terms, than improving from 70% to 95% intactness. Yet both have the same arithmetic difference. For this reason, most people would use the percentage difference, not the simple arithmetic difference.

Again, this section gives no information on the baseline against which a "keep it below two degrees" strategy could be compared, in terms of biodiversity outcomes.

To add to the confusion, S3 figure labels the benefit axis as "% benefit", rather than the arithmetic difference described everywhere else.

*The cost-effectiveness of each strategy i (CEi) was calculated as the expected benefit (sum of*

*876 all taxa) divided by the expected cost (Ci), where expected benefit was calculated as potential*

*877 benefit (Bi) multiplied by feasibility (Fi):*

*𝐶𝐸𝑖 =𝐵𝑖𝐹𝑖 /𝐶𝑖*

when...

*Feasibility consisted of two aspects:*

*718 Likelihood of uptake was estimated for each action and represents the likelihood that*

*719 policymakers will agree to implement this action*

*...*

*Likelihood of success*

*722 was estimated per strategy and represents the likelihood that the strategy will meet its*

*723 objectives, assuming that all actions have been successfully implemented.* ***Overall feasibility***

***724 per strategy was calculated as a product of the two likelihoods and is provided as a probability***

*725 (S3 Fig.; S1 Data).) [My emphasis].*

I'm afraid this seems to be a nonsense. If cost effectiveness is benefit times feasibility over cost, and feasibility is the product is the likelihood of uptake and the likelihood of success, then a strategy that is not fully taken up should not have a full cost associated with it! And yet, the cost-effectiveness measures seem to compare the full cost of the proposed strategy against the benefits of a partial implementation of it? If costings assume the action was implemented in full, then benefits should do so as well in a CBA.

This also raises the question: why, in the section immediately preceding those lines, is the benefit of an intervention apparently calculated as the simple change in intactness (**without** weighting by likelihood of success), and then recalculated **with** the weighting in the cost effectiveness section?

Even more confusing, the next section (the complementarity analysis) then seems to add in the weighting again:

*The expected intactness*

*913 𝑃𝑖𝑗 for each taxon j under a given strategy i can be calculated as follows:*

*𝑃𝑖𝑗 = 𝑃 ̅ 𝑏𝑎𝑠𝑒𝑙𝑖𝑛𝑒𝑗 + 𝐹𝑖𝐵 ̅ 914 𝑖𝑗,*

In other words, the benefit of the intervention is weighted by the feasibility of the intervention on this occasion.

*917 𝐵 ̅ 𝑖𝑗 [is] the mean potential benefit across*

*918 contributing experts of applying strategy i for taxon j.*

Again, this seems inconsistent. If this measures the benefit of applying the strategy, then it seems odd to state that you are multiplying it by the likelihood of NOT applying the strategy. This could be more precisely worded.

In summary, this all seems inconsistent and would either need to be calculated consistently, or to explain (for example) why the authors are fully costing strategies that are not going to be fully implemented.

Additionally, how fair is it to assume that the current conservation strategies (the baseline) will retain 100% of their "feasibility" over the next 83 years, whereas the alternative intervention is downgraded by its feasibility? Were the experts instructed to consider possible changes in the regularity of implementation or the possibility of current conservation becoming less/more successful between now and 2100? Or is the BAU intactness score simply a guess about biodiversity in 2100 based on current conservation activities, their success and their uptake rates? Does that guess even consider the feasibility of the current activities? Behavioural economics would suggest that practitioners engaged in current activities may have a biased appreciation of their likely future success.

**Complementarity section: major question**

*Conveniently the 𝑃𝑖𝑗 can be assessed*

*919 under different persistence thresholds and provide a binary matrix T such that an element 𝑇𝑖𝑗*

*920 takes value 1 if the expected persistence of taxon j under strategy i (𝑃𝑖𝑗) is above the designated threshold and 0 otherwise.*

*The complementarity problem is usually formulated as maximising the number of taxa above the designated threshold for a given budget (Carwardine et al. 2019). Here, we chose to minimise the cost of securing a given number of taxa above threshold.*

*The first set of decision variables 𝑦𝑖 determines if a strategy 𝑖 ∈ 𝑆 is selected (𝑦𝑖 = 1) or not (𝑦𝑖 = 0).*

*If strategy i is selected, the taxon j with values of 𝑇𝑖𝑗=1 is assumed secured. The second set of*

*auxiliary decision variables 𝑥𝑖𝑗 identifies the taxon j secured by strategy i – these decision*

*variables ensure we reach the target of a given number of taxa to secure (identified by Target).*

*(c2): For the purpose of the optimisation, only one strategy can count towards securing*

*a taxon above the threshold.*

This is a somewhat odd specification of the problem, again. To reiterate: if the conservation benefit is multiplied by feasibility, and feasibility includes the likelihood of not implementing the strategy, then it would again seem that the optimized investment profile currently includes paying for things which were not actually done!

Perhaps more important is the way the specification seems to implement the measures one at a time and then assume that species intactness only changes as a result of the implemented strategy. (Thus, in the first step in Figure S5, it seems that only "controlling human activities" would be implemented). But does this take account of the negative impact of inaction on the other fronts. For example, if "control human activities" is implemented, the program calculates that a large number of taxa will achieve a certain intactness threshold based purely (in my reading) on the benefit of controlling human activities. But if the same taxa are likely to lose intactness from e.g. invasives, then intactness would correctly be calculated as the net outcome, including both the benefit of the implemented action and the losses occurring due to any non-implemented action. Thus, I suspect the correct specification would be not 'expected persistence under strategy i', but rather 'expected persistence under the full suite of strategies, including those not implemented', i.e. sum of all Pij where some strategies are set to a marginal zero. If my description is not the correct one, I would like to see some text clarifying how the net impact of all actions and inactions is being calculated. Is it that the "cost of inaction" is implicitly contained in the baseline (as I suspect is happening)? If so, then please clarify that inaction on some fronts negatively affects intactness at the same time that action on other fronts positively affects it.

Additionally, there is a clash between what many readers will assume is "complementarity" in conservation actions, and what the integer linear programming procedure means by the word. For many readers, "complementary" actions might reinforce each other, making them more effective, but the ILP process described does not seem to incorporate such "sum of the whole is greater than the parts" effects, despite the text referring to them (e.g. action achieving climate improvement would reduce the need for action on invasives). There will also be some redundancy, for example if protected areas contribute to vegetation conservation. Quite a lot more needs to be done by the authors here, either to account for how multiple conservation actions work together in the modelling, or to discuss how a failure to account for that could influence the conclusions.

**Discounting of future costs (but not discounting of future benefits?): major question/revision:**

As far as I can make out, the annual costs are calculated over exactly 83 years and then suddenly are set to zero in 2100. Moreover, it seems as if discounting has been applied to future costs but not to future benefits. (Benefits are essentially the delta intactness assumed achieved by a strategy in 2100). Typically, economists discount the future flow of benefits, rather than costs but not benefits. It is therefore odd to discount something not normally discounted, not discount something that is normally discounted, and then compare the two anyway.

The first question here, however, is whether applying a discount rate to future costs is appropriate, unbiased or even useful at all. On appropriateness, discounting future costs assumes that an investor in conservation is looking for a financial return, which is unusual for an Antarctic conservation situation. Government costing exercises typically want to know the simple cost, not a discounted cost, because future discounting of costs would be such an odd thing to do. It's therefore not clear how appropriate the future discounting of cost is, on principle.

As regards to bias, it would appear that the authors describe a "social discount rate". Again, social discount rates are usually applied to benefits, including non-financial benefits such as future outcomes for biodiversity. But the cost-benefit analysis is clearly biased if one discounts the costs but not the benefits.

As regards to usefulness: if the main analysis is about the relative cost, cost effectiveness and cost complementarity of the various strategies, then the outcome will be the same irrespective of how these are discounted, unless there are major differences in how costs unfold over future years. The future trajectory of costs is neither described by the authors, nor justified as a reason for the discounting. This would need to be remedied before a reviewer could even make a judgement on whether discounting of costs was genuinely useful.

If cost discounting seems relatively uninformative, then this raises a concern that the actual cost is vastly understated because by 2100, costs presumably fall to almost zero (after 83 years of compound discounting at 2%*)*, which would be a most questionable conclusion. This indeed becomes apparent when the authors mention, in a brief parenthesis, that without discounting, the cost would jump from $1.92 billion to $3.69 billion i.e. it would pretty much double.

I do rather suspect that it is the question of how to spread out capital costs that influences some of the authors' thinking around discounting. But another way to spread capital cost is to assume amortization on, effectively, a loan to cover initial set-up costs for a strategy.

Overall, I would say that the authors either need to discount future benefits and future costs in a consistent way, or they could quite feasibly drop the discounting of future costs completely, since it is atypical and of unclear usefulness in the comparisons (e.g. the ratio of two discounted values is similar to the ratio of two undiscounted values, and most of the paper is about such ratios).

**Future values in general and setting costs to zero after exactly 83 years.**

On a broader point: how can we possibly estimate the costs of actions up to 83 years in the future? Did the authors and experts largely assume current costs, ignoring how these would change in the future? For example, if climate change causes more invasive species in the second half of the century, that would increase the future costs of combating invasives. And if human population and wealth increase (as is likely), then both tourism and other pressures would increase over time. Any such future change would alter the outcomes of all the cost and cost effectiveness analyses performed here, but that possibility does not seem to be addressed in the sensitivity testing.

**Minor points**

*However, for*

*303 those species that are predicted to benefit with climate change, at least initially (e.g. gentoo*

*304 penguins), they benefit less with the milder RCP2.6 scenario in contrast to RCP4.5 or RCP8.5.*

*17*

*Thus, it appears that they have reduced intactness 305 with the RCP2.6 ‘Influence external policy'*

I realise that counterintuitive results need to be explained but why not present the main patterns, then at the end, say that some results may seem surprising e.g. Gentoo penguins doing better and better and climate change gets worse and worse, and explain what lies behind those surprises. The way the text structure is set up currently, it feels like the anomalous is being given the same weight as all the main results.

*337 Considering multiple social discount rates, where future costs are discounted to present-day*

*338 values (PVs; see Materials and Methods), combining all strategies was consistently estimated*

*339 to be the most expensive strategy*

Unless different discounting rates were applied to different strategies, then the comparative cost of the strategies has no connection to the discount rates. The start of this sentence therefore seems to be a red herring.

*Line 376*: the first paragraph clearly belongs to the previous section on cost, not to the new section on cost effectiveness. Overall, there still seems to be some work to be done on clearly structuring the manuscript. The conclusions are important, so it is really worth getting this right!

*Section on Implementing the Conservation Actions:*

This is well written, eminently sensible and should provide the core of what this paper is about. Perhaps a mention of the problems of financing a solution when one is not dealing with a single nation's sovereign territory would be a useful addition

*the strategies were reasonably robust*

*519 to all sources of uncertainty.*

Not so, and this is unscientific. They are robust to precisely the uncertainties that the authors tested, no more. It would be more useful and honest to state at this point in the ms what uncertainties were tested for, precisely.

*522 despite the uncertainties in our experts estimates,*

an apostrophe is needed here

*the top three most*

*523 beneficial, or most cost-effective strategies are the same regardless of RCP, region or whether*

*524 upper or lower bound is used (see Fig. 1; Table 2; S5, S6 Figs.; S4, S5 Tables).*

Why not restate them briefly? Indeed, by this point, I had no memory of what the "top three most beneficial strategies" might have been - except that reducing climate change would be most important.

*611 2100 (UNFCCC 2015). Therefore, RCP2.6, the low emissions scenario and the only one to*

*612 keep global warming to <2 °C (IPCC 2013; Gasser et al. 2015), was used to assess the*

*613 ‘Influence external policy’ and ‘All Strategies Combined’ strategies*

Does this mean that the experts were told to assume that the action 'influencing external (global) climate policy' would lead to the earth limiting climate change to a maximum two-degree temperature increase globally, and to estimate how much more intact the biota would be if we achieved that? If so, what is the baseline against which the intactness was measured? How are the benefits calculated?

Later, the text says *"if.. the world were committed to >2 °C warming".* This is highly imprecise and does not give a quantitative baseline against which to compare. How much warming above two degrees? It makes a lot of difference.

*764 it was assumed that costs and feasibility would remain the same for*

*765 both RCP4.5 and RCP8.5.*

This seems like a poor assumption, requiring more justification and more exploration of the consequences of this decision.

*S4 Figure* and elsewhere: it's confusing to label the cost as "present value". Please label it as "cost" and if necessary, use brackets after that to indicate that the cost has been discounted. Also, these figures seem to have returned to using the total cost (a total in which annual spending is arbitrarily halted after exactly 83 years) that was the subject of an objection in the previous review.

If the authors indeed wish to apply a discount rate to future annual costs (something that is more typically applied to benefits), then at minimum, they need to specify in all figures whether they are using the dollar values for a particular year (constant dollars 20XX). Otherwise, inflation makes a mockery of the discounting.

*S5 Figure*: here it suddenly becomes apparent that a cost, and a cost effectiveness, has been placed upon "influence external policy" - something that after three readings, I had deduced had not been done because it would not remotely be a fair or unbiased comparison. Examination of the data table shows that this cost largely involves employing a phalanx of communications or back-office professionals to influence COP discussions and public opinion on climate and plastics. When this is put into a complementarity graphic, it really brings home how heroic the assumptions have to be to make this work: expected cost effectiveness is investment divided by likely degree of success, but what is the likelihood of success for influencing of global COP outcomes? In other words, is it even remotely reasonable to estimate the impact of communicators on a global climate goal (ignoring all the other parties trying to achieve similar things), and then confidently calculate their likely cost effectiveness? My own feeling is that no, it is not reasonable to make such giant leaps of faith in assuming what the communicators could achieve for global climate outcomes.

In the graphic itself, the discontinuous steps need more explanation. For example, "new infra + protecting areas" seems to cover a range of possible budgets from about $200 million to about $800 million, which is likely to be highly confusing to the reader.

There is a surprising different between RCP4.5 and RCP8.5, in that protecting areas is an efficient strategy in one but not the other. This also requires explanation.

*lower or upper bounds for the cost or feasibility estimates were not*

*973 elicited.*

This seems quite an unusual omission, especially for budget estimation. Moreover, it seems slightly inconsistent with the online material, where it is stated:

*" Cost Range
A number of actions have a cost range depending on various factors outlined in the action details/ assumptions made (eg. the potential number of invasive species
that could establish, or whether it will take 2 or 10 FTE for a job). When a range is presented in the cost column, it is the range of minimum-maximum price
expected for that action"*

*S1Data:*

The table should be given a table identifier (e.g. Table S1).

If there are two likelihoods in the feasibility score, why is only one (likelihood of uptake) included in the table, and the arguably more interesting one (likelihood of success in having the expected impact) is omitted?
